# Supplementary figures and images for: Intragenomic variation in non-adaptive nucleotide biases causes underestimation of selection on synonymous codon usage
Source: PLoS Genet. 2022 Jun 17;18(6):e1010256. doi: 10.1371/journal.pgen.1010256 (PMC9246145; doi:10.1371/journal.pgen.1010256)

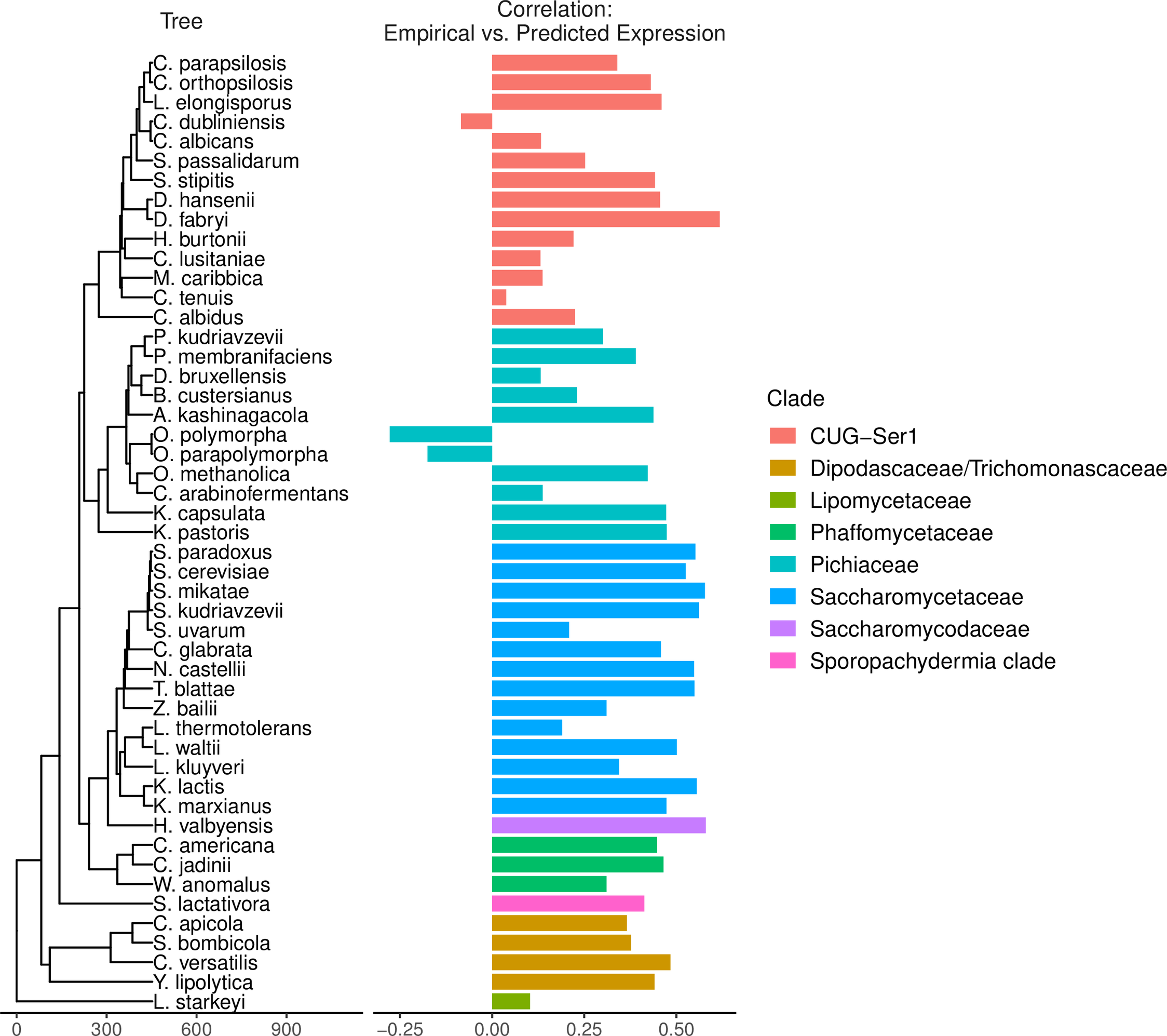

Supplement: S1 Fig — Spearman rank correlation coefficients comparing predicted gene expression estimates from ROC-SEMPPR ConstMut fit with empirical estimates of mRNA abundance from RNA-seq data across 49 Saccharomycontina budding yeasts. (TIF) [file pgen.1010256.s001.tif]

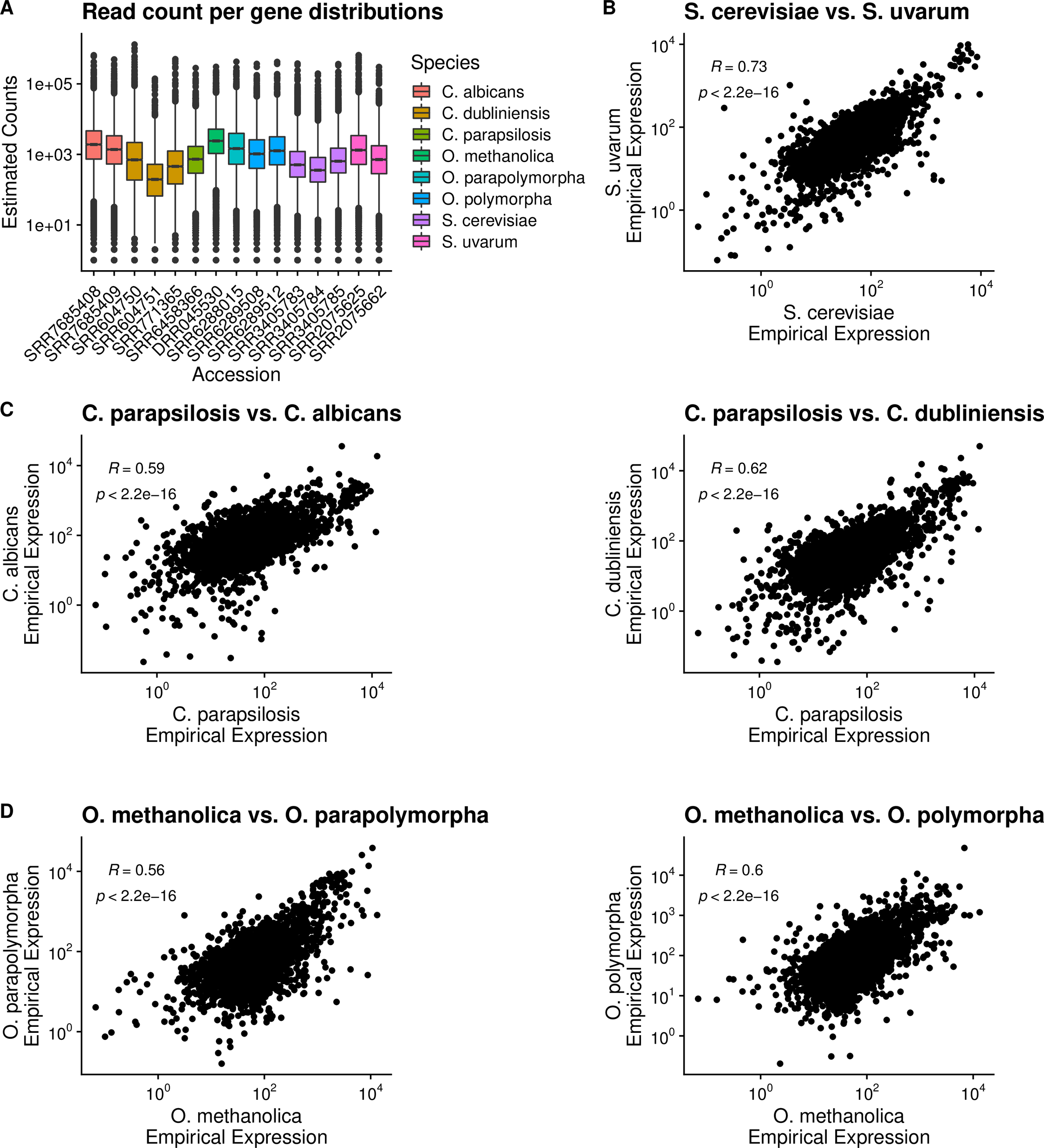

Supplement: S2 Fig — (A) Distribution of per-gene estimated counts from kallisto. (B-D) Comparison of empirical expression estimates across species using one-to-one orthologs. Correlations represent Spearman rank correlation coefficients. (TIF) [file pgen.1010256.s002.tif]

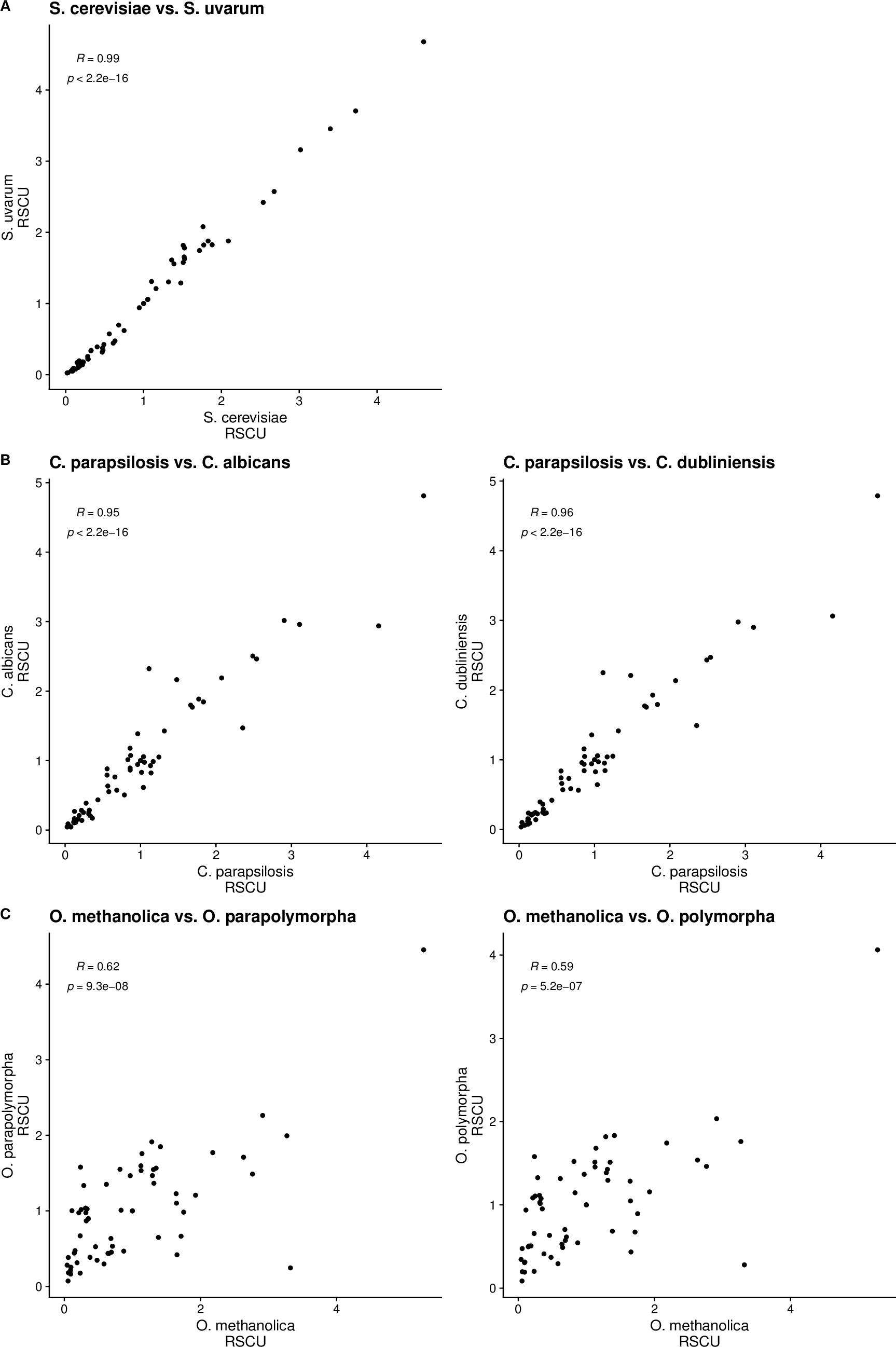

Supplement: S3 Fig — Across-species comparison of RSCU values calculated from the most highly expressed genes (top 5% based on empirical expression estimates). (A) Saccharomyces. (B) Candida. (C) Ogataea. Correlations represent Spearman rank correlation coefficients. (TIF) [file pgen.1010256.s003.tif]

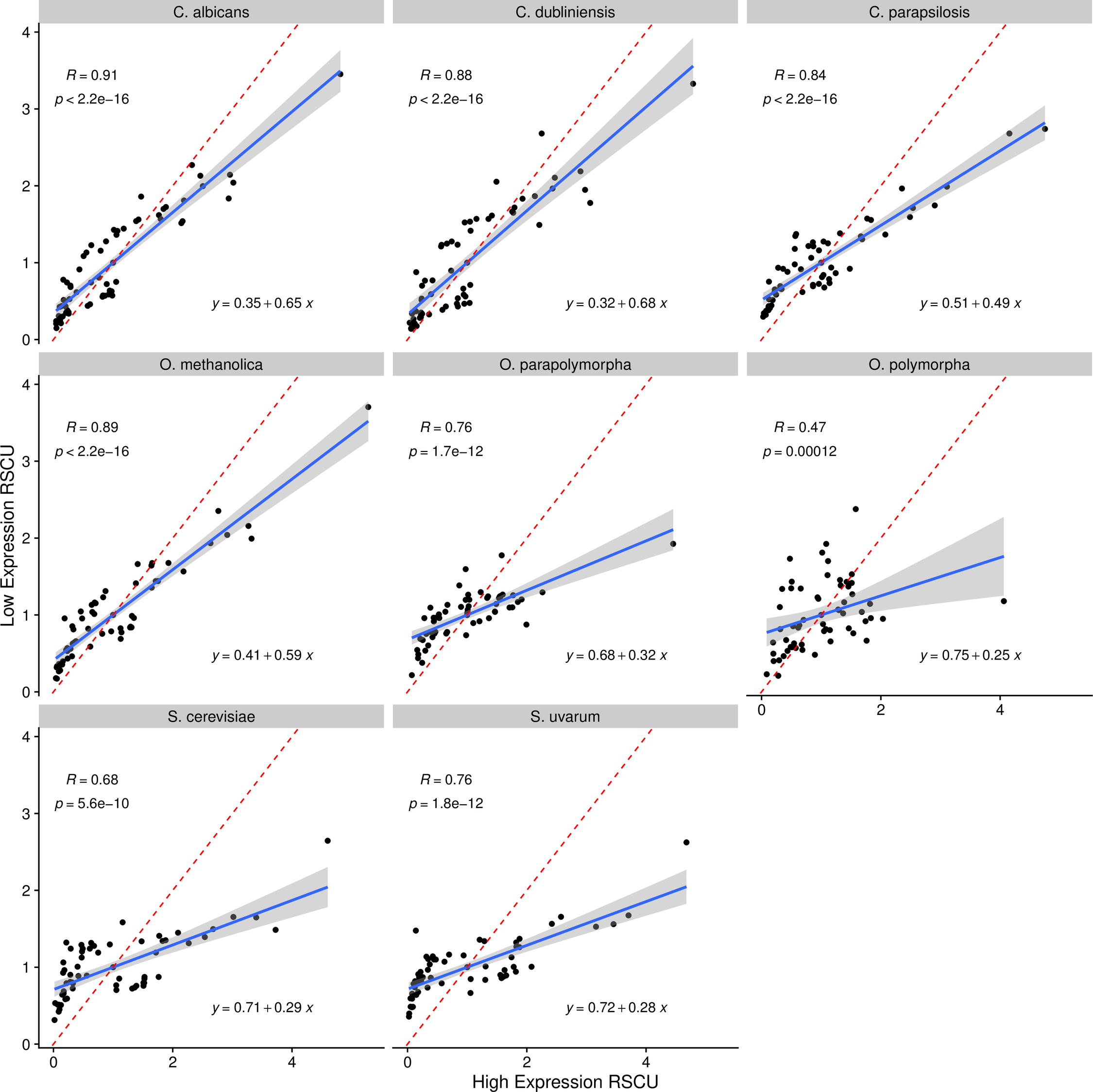

Supplement: S4 Fig — Comparison of RSCU values calculated from most highly and lowly expressed genes (top 5% and bottom 5% of expression estimates) for each species. Correlations represent Spearman rank correlation coefficients. (TIF) [file pgen.1010256.s004.tif]

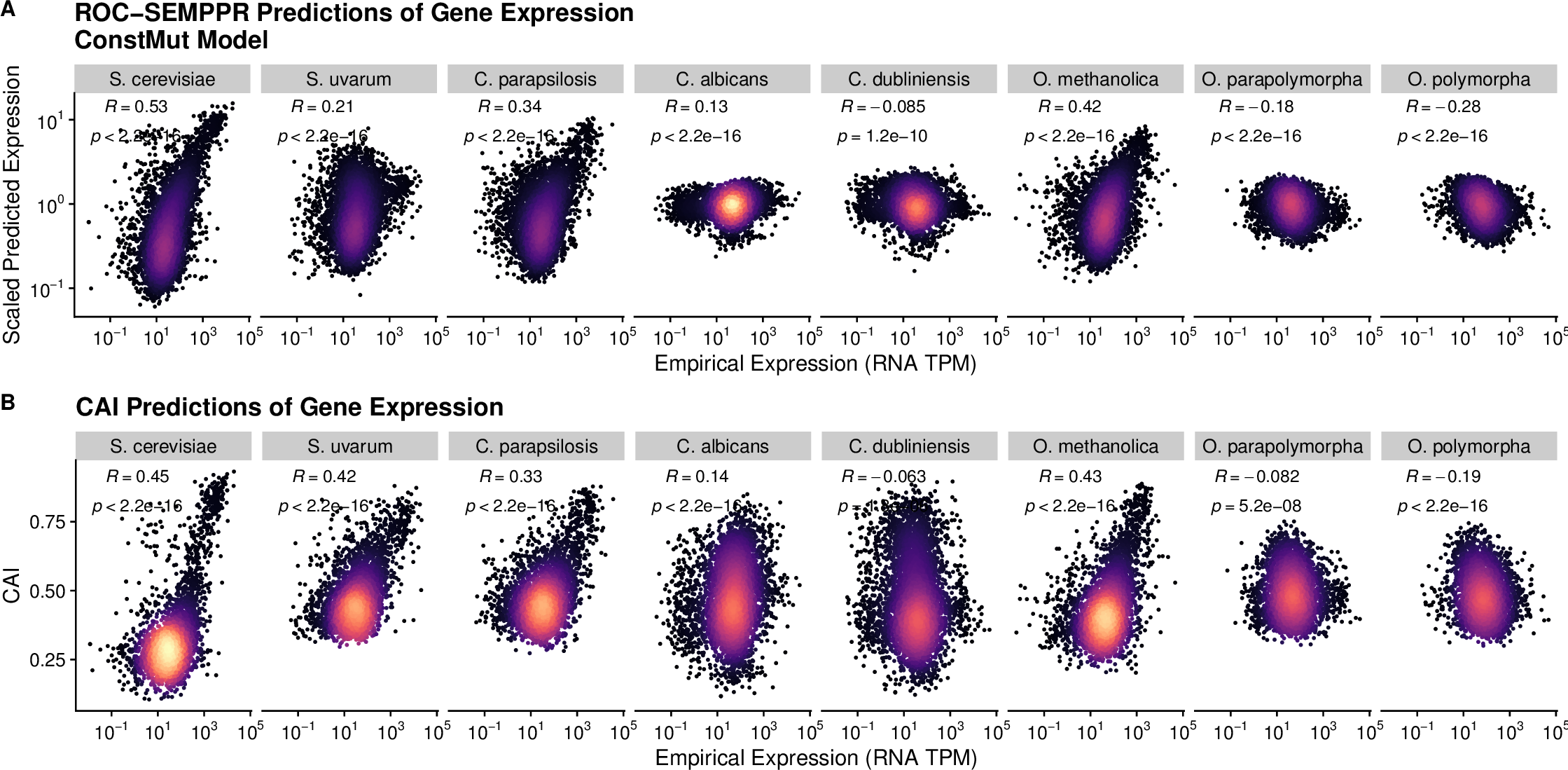

Supplement: S5 Fig — Comparing ability to predict expression using (A) ROC-SEMPPR (ConstMut) and (B) Codon Adaptation Index (CAI). CAI was estimated using CodonW, with the correspondence analysis built-in to CodonW used to identify the reference set. (TIF) [file pgen.1010256.s005.tif]

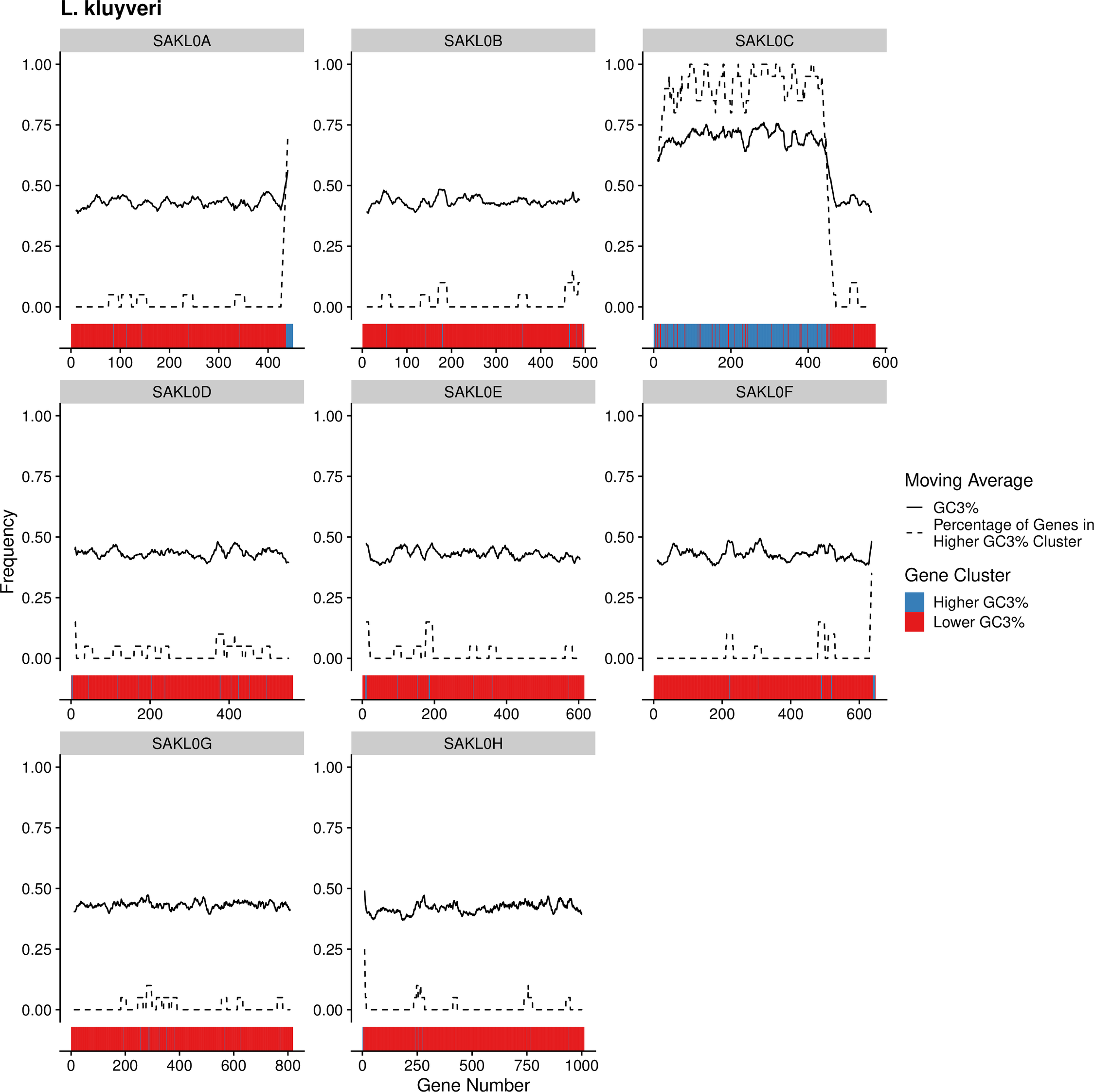

Supplement: S6 Fig — Per-gene GC3% content across all L. kluyveri chromosomes quantified as a moving average using a 20 gene sliding window (solid line). For each 20 gene window, the percentage of genes assigned to the Higher GC3% regime is also shown (dashed line). Color bars indicate the mutation regimes for Higher and Lower GC3% (blue and right, respectively). The region of high GC3% content on chromosome SAKL0C is the result of an introgression. (TIF) [file pgen.1010256.s006.tif]

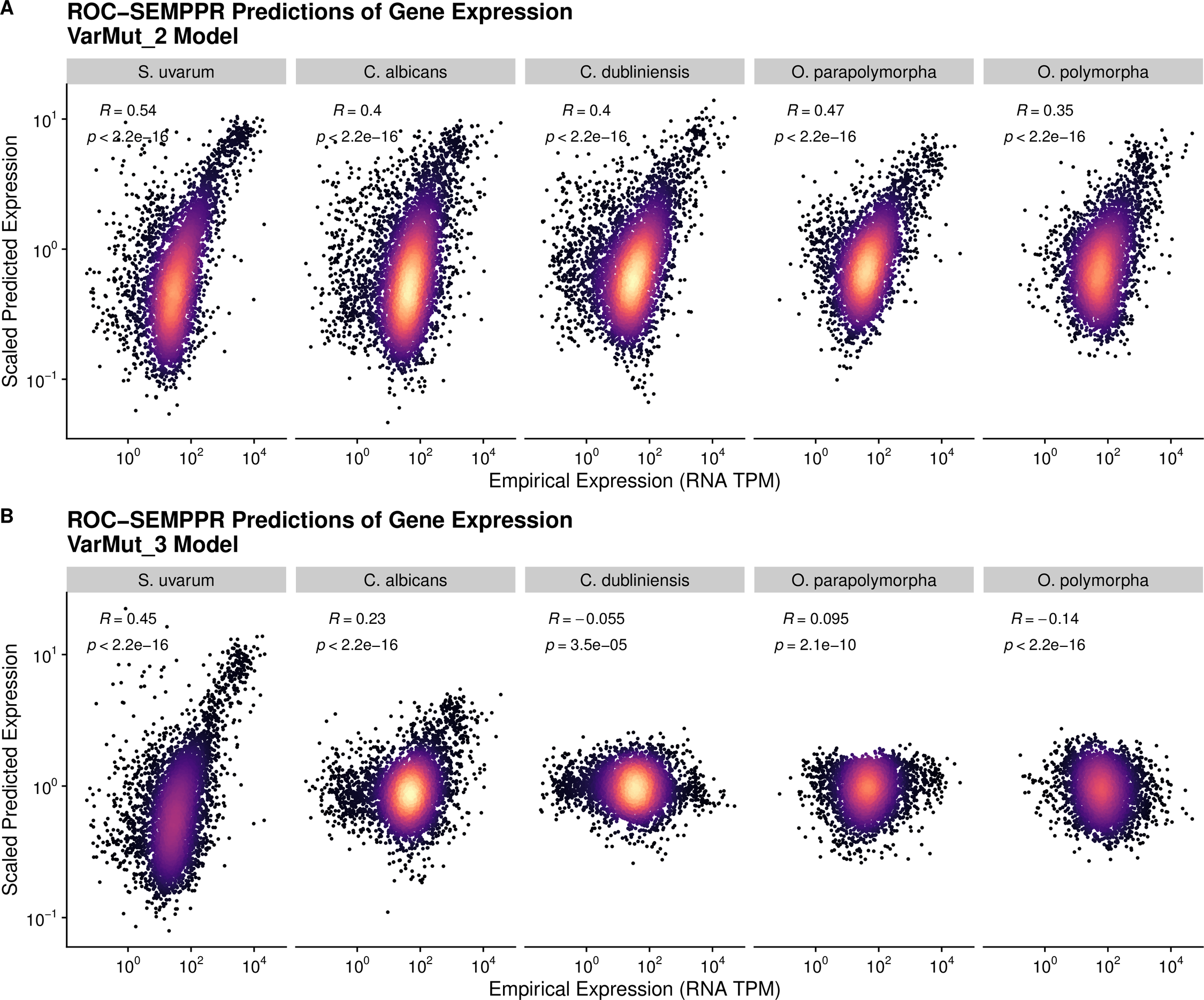

Supplement: S7 Fig — (A) VarMut_2 model using k = 2 clusters. (B) VarMut_3 model using k = 3 clusters. (TIF) [file pgen.1010256.s007.tif]

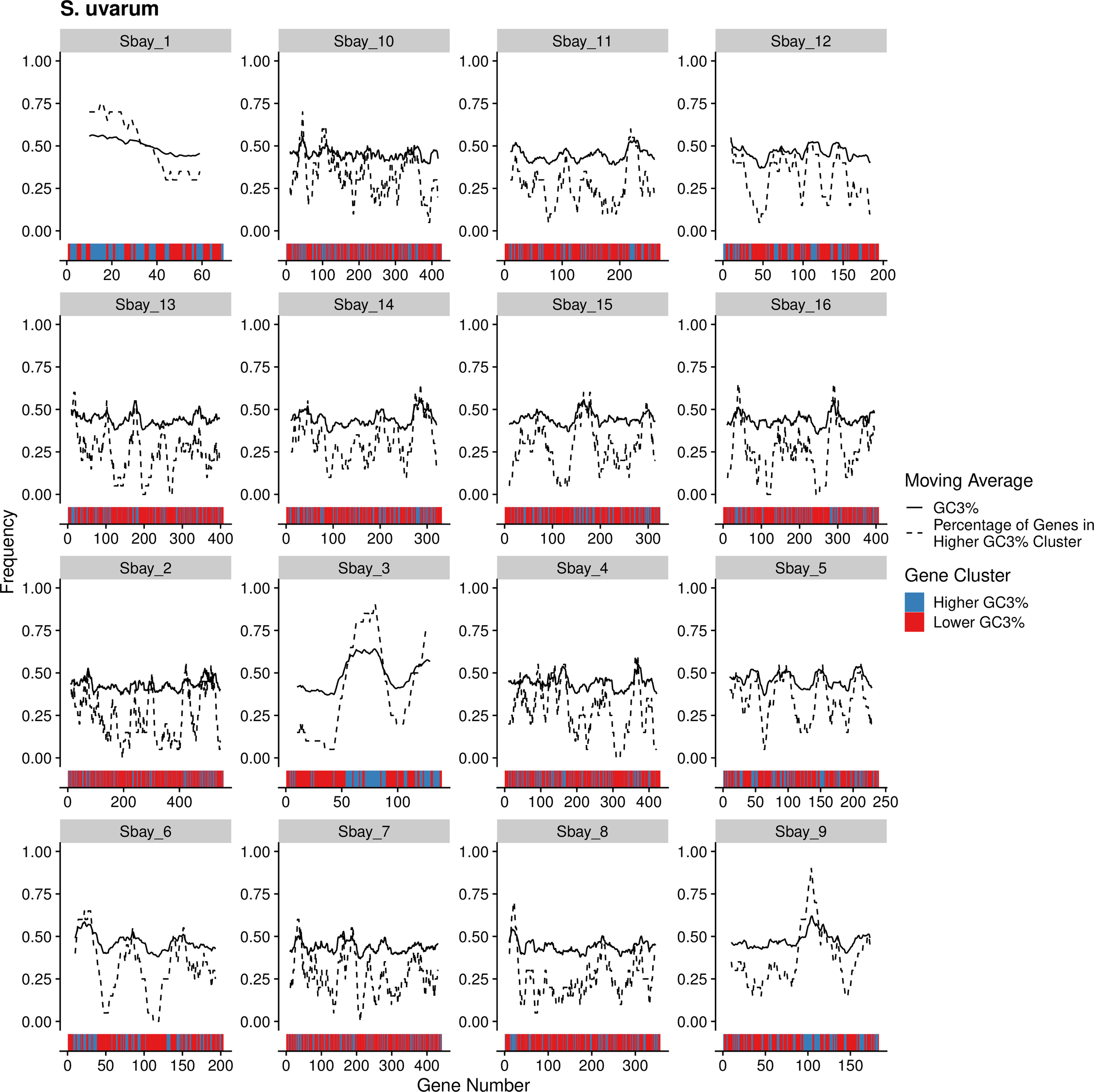

Supplement: S8 Fig — Per-gene GC3% content across all S. uvarum chromosomes quantified as a moving average using a 20 gene sliding window (solid line). For each 20 gene window, the percentage of genes assigned to the Higher GC3% regime is also shown (dashed line). Color bars indicate the mutation regimes for Higher and Lower GC3% (blue and right, respectively). (TIF) [file pgen.1010256.s008.tif]

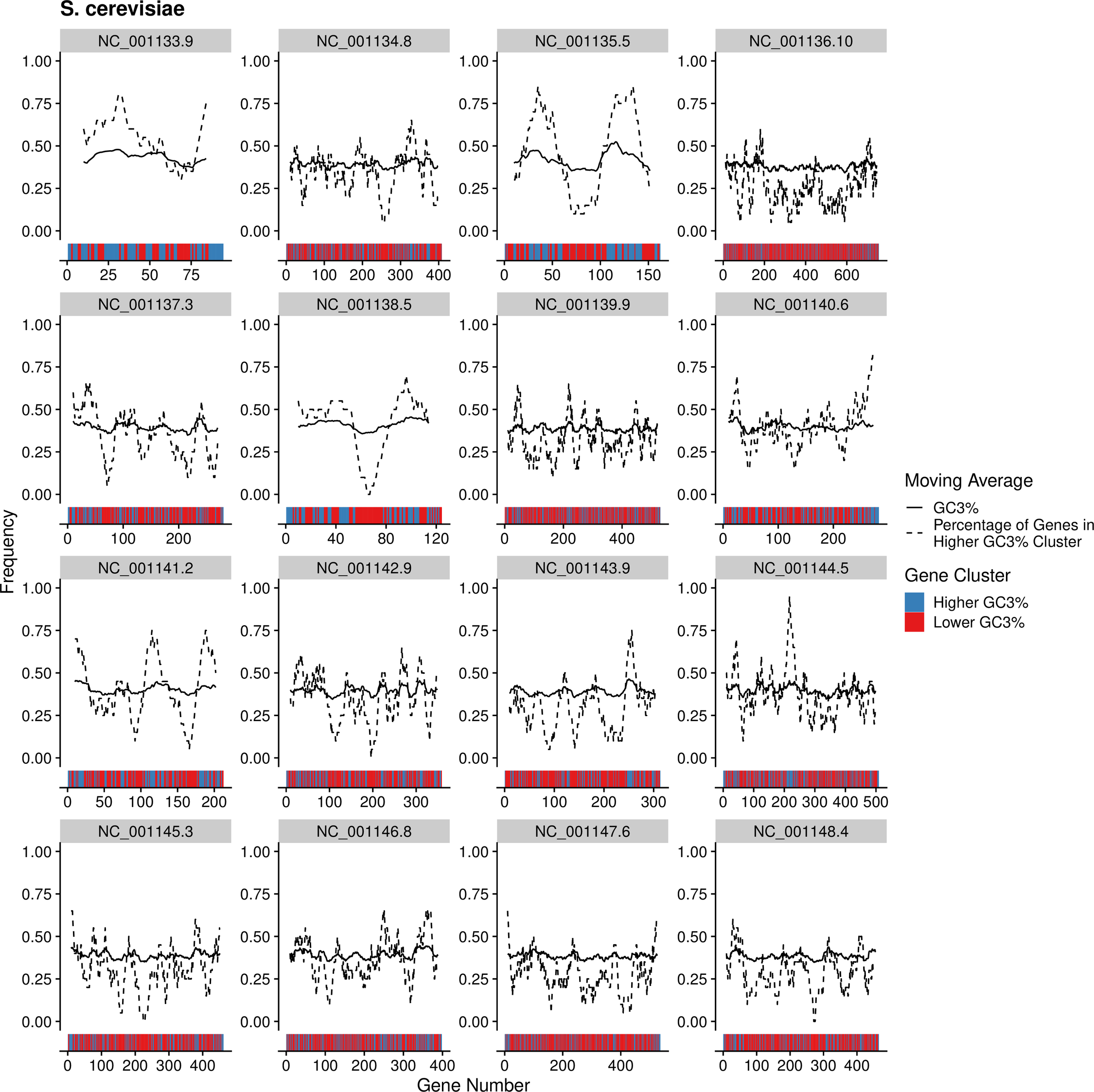

Supplement: S9 Fig — Per-gene GC3% content across all S. cerevisiae chromosomes quantified as a moving average using a 20 gene sliding window (solid line). For each 20 gene window, the percentage of genes assigned to the Higher GC3% regime is also shown (dashed line). Color bars indicate the mutation regimes for Higher and Lower GC3% (blue and right, respectively). (TIF) [file pgen.1010256.s009.tif]

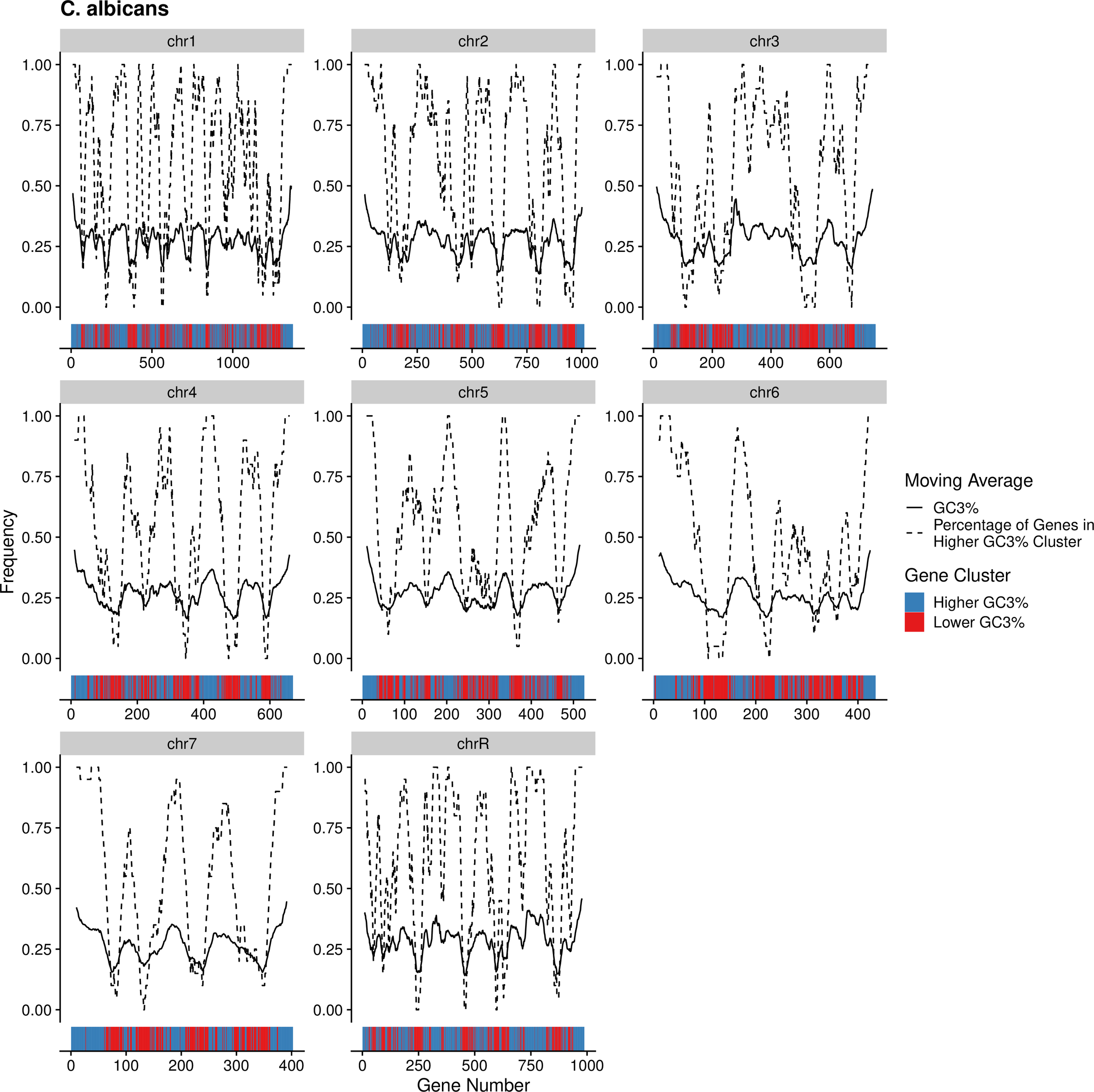

Supplement: S10 Fig — Per-gene GC3% content across all C. albicans chromosomes quantified as a moving average using a 20 gene sliding window (solid line). For each 20 gene window, the percentage of genes assigned to the Higher GC3% regime is also shown (dashed line). Color bars indicate the mutation regimes for Higher and Lower GC3% (blue and right, respectively). (TIF) [file pgen.1010256.s010.tif]

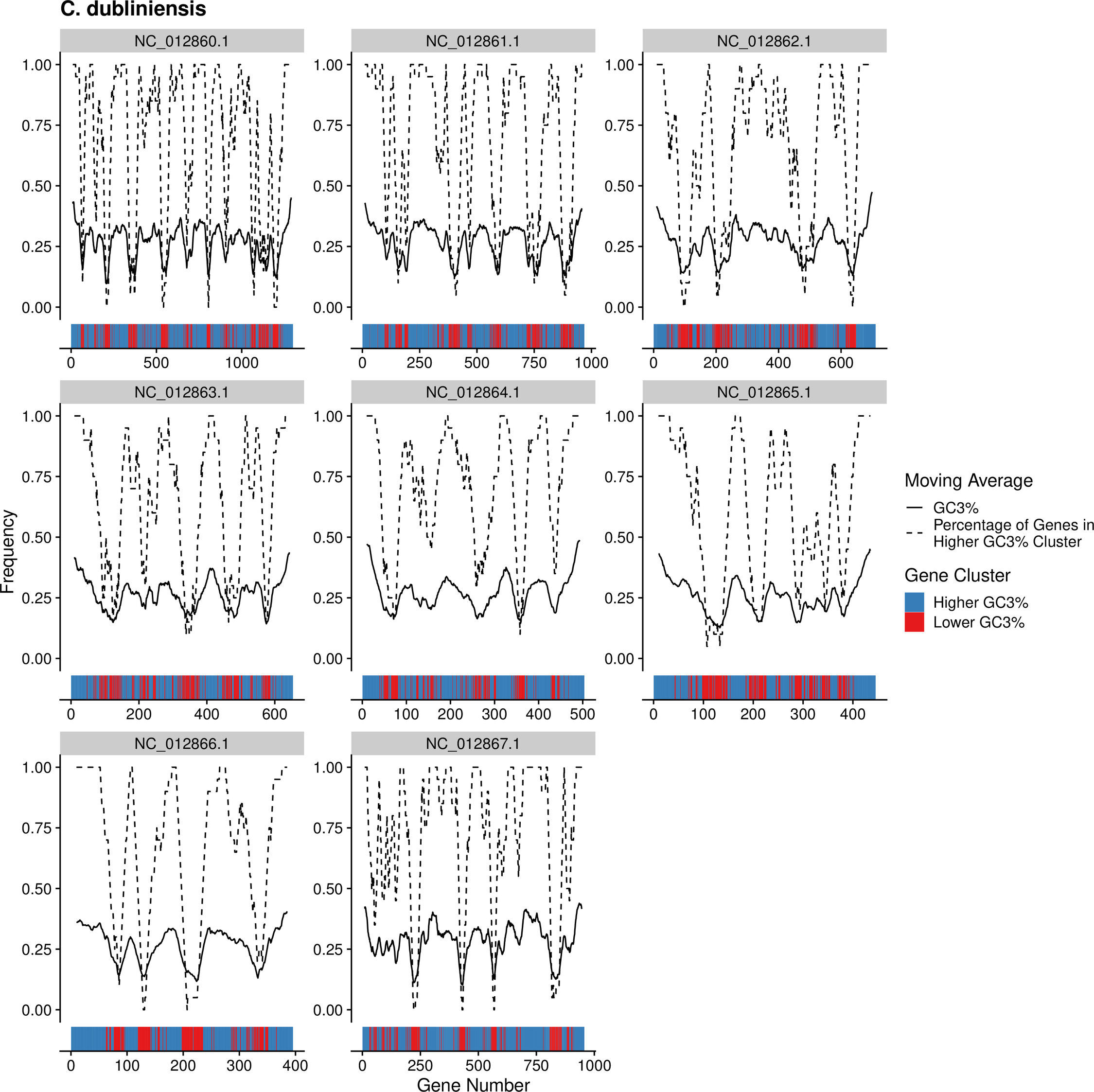

Supplement: S11 Fig — Per-gene GC3% content across all C. dubliniensis chromosomes quantified as a moving average using a 20 gene sliding window (solid line). For each 20 gene window, the percentage of genes assigned to the Higher GC3% regime is also shown (dashed line). Color bars indicate the mutation regimes for Higher and Lower GC3% (blue and right, respectively). (TIF) [file pgen.1010256.s011.tif]

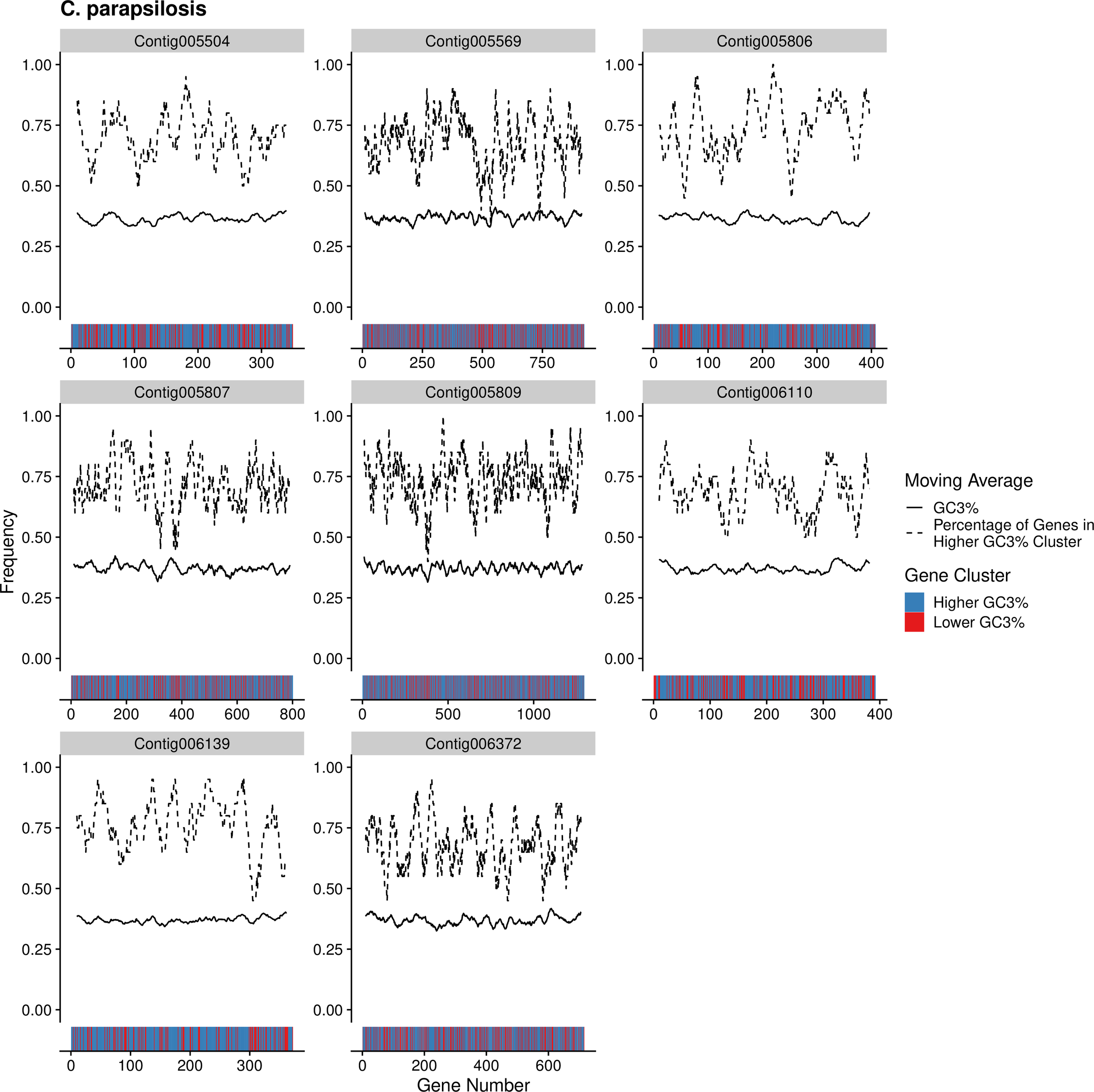

Supplement: S12 Fig — Per-gene GC3% content across all C. parapsilosis chromosomes quantified as a moving average using a 20 gene sliding window (solid line). For each 20 gene window, the percentage of genes assigned to the Higher GC3% regime is also shown (dashed line). Color bars indicate the mutation regimes for Higher and Lower GC3% (blue and right, respectively). (TIF) [file pgen.1010256.s012.tif]

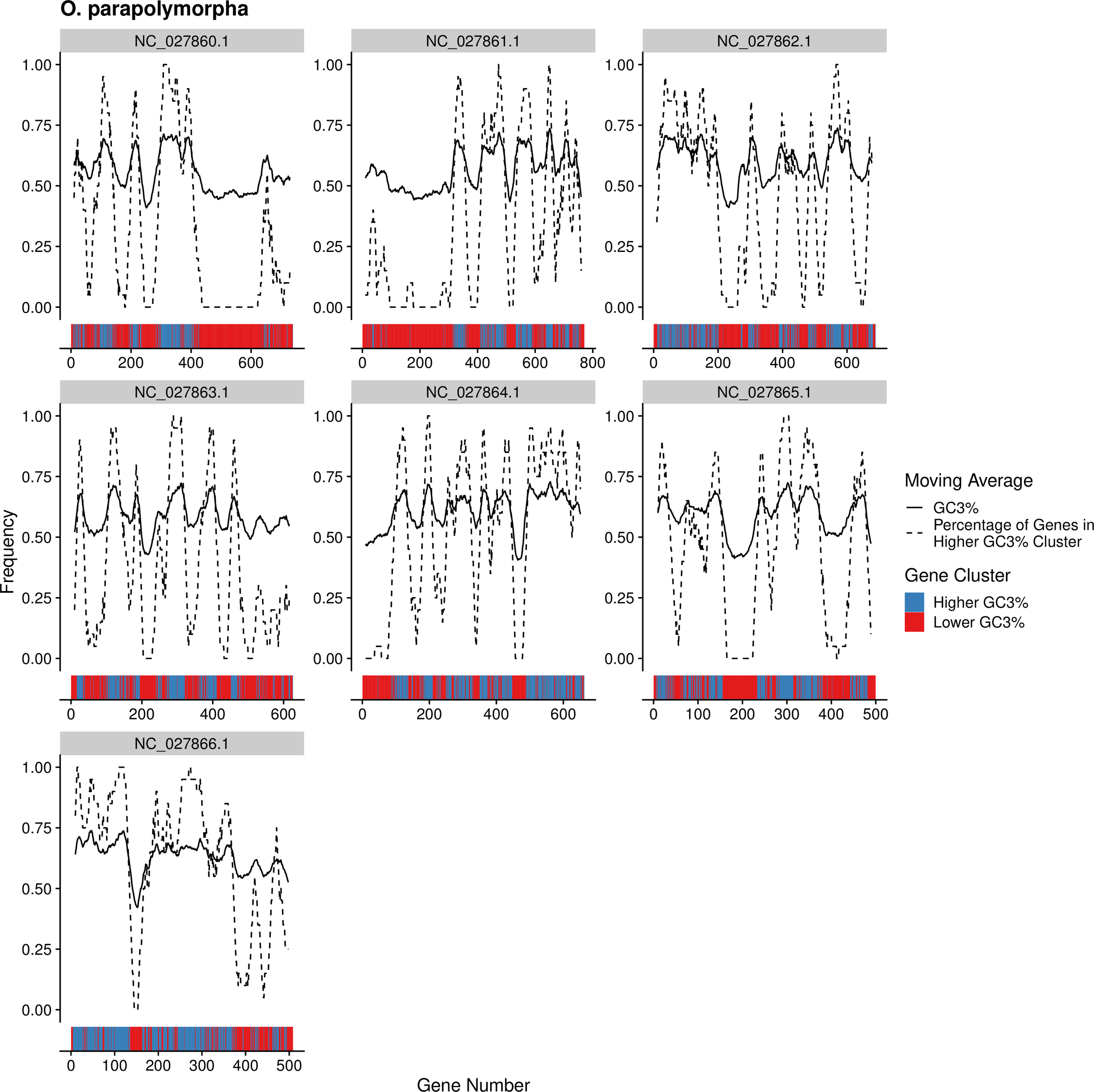

Supplement: S13 Fig — Per-gene GC3% content across all O. parapolymorpha chromosomes quantified as a moving average using a 20 gene sliding window (solid line). For each 20 gene window, the percentage of genes assigned to the Higher GC3% regime is also shown (dashed line). Color bars indicate the mutation regimes for Higher and Lower GC3% (blue and right, respectively). (TIF) [file pgen.1010256.s013.tif]

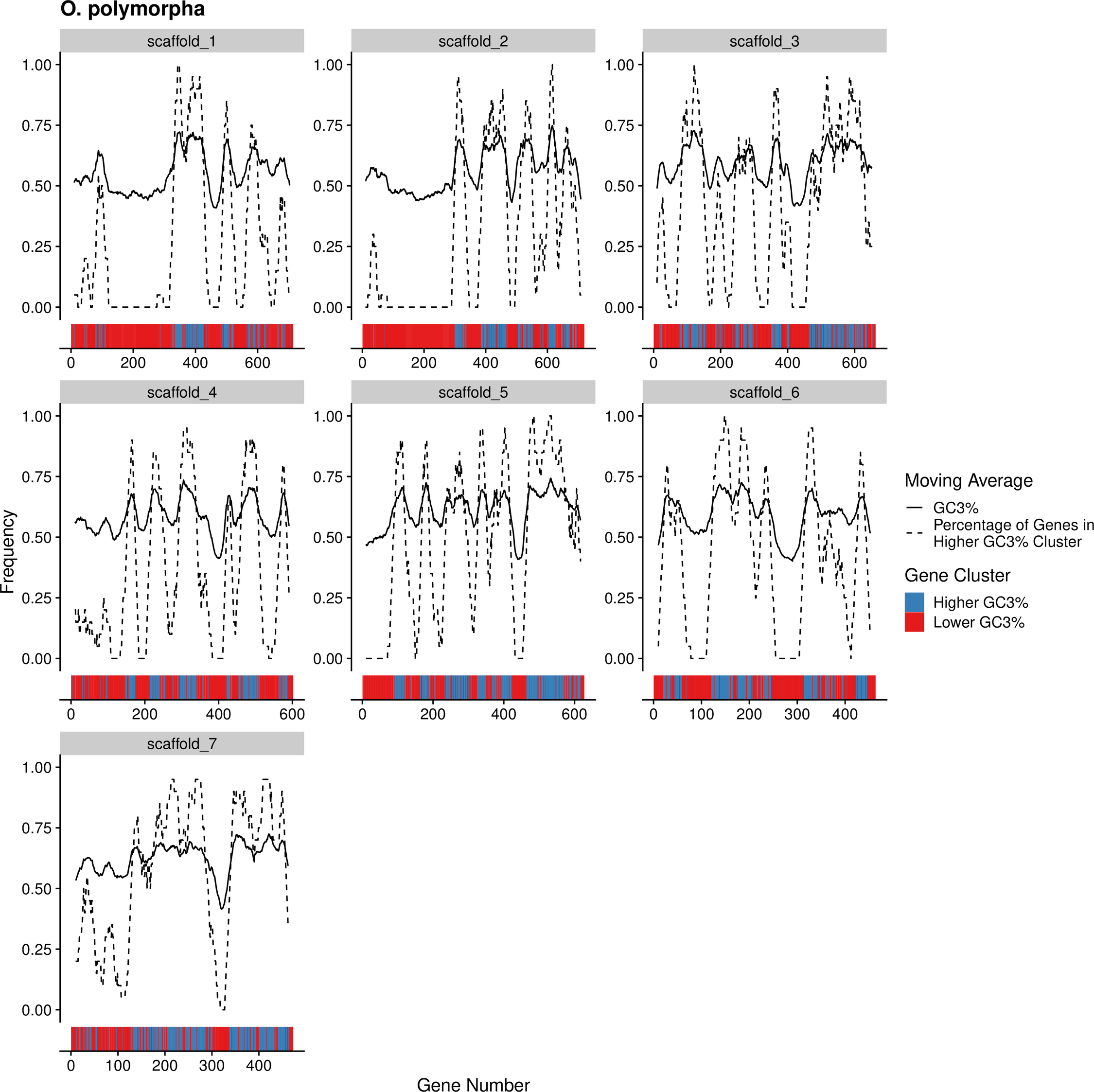

Supplement: S14 Fig — Per-gene GC3% content across all O. polymorpha chromosomes quantified as a moving average using a 20 gene sliding window (solid line). For each 20 gene window, the percentage of genes assigned to the Higher GC3% regime is also shown (dashed line). Color bars indicate the mutation regimes for Higher and Lower GC3% (blue and right, respectively). (TIF) [file pgen.1010256.s014.tif]

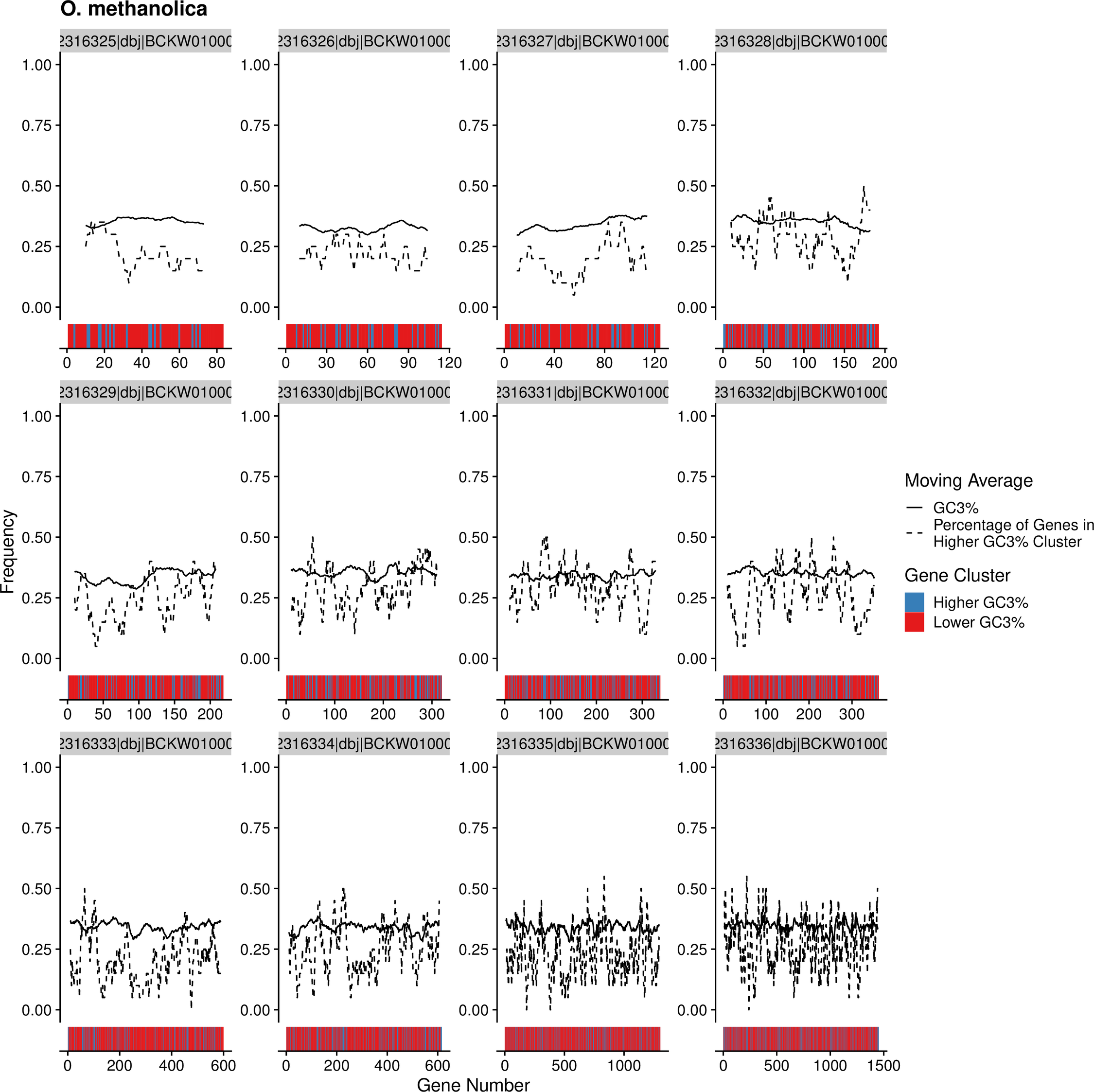

Supplement: S15 Fig — Per-gene GC3% content across all O. methanolica chromosomes quantified as a moving average using a 20 gene sliding window (solid line). For each 20 gene window, the percentage of genes assigned to the Higher GC3% regime is also shown (dashed line). Color bars indicate the mutation regimes for Higher and Lower GC3% (blue and right, respectively). (TIF) [file pgen.1010256.s015.tif]

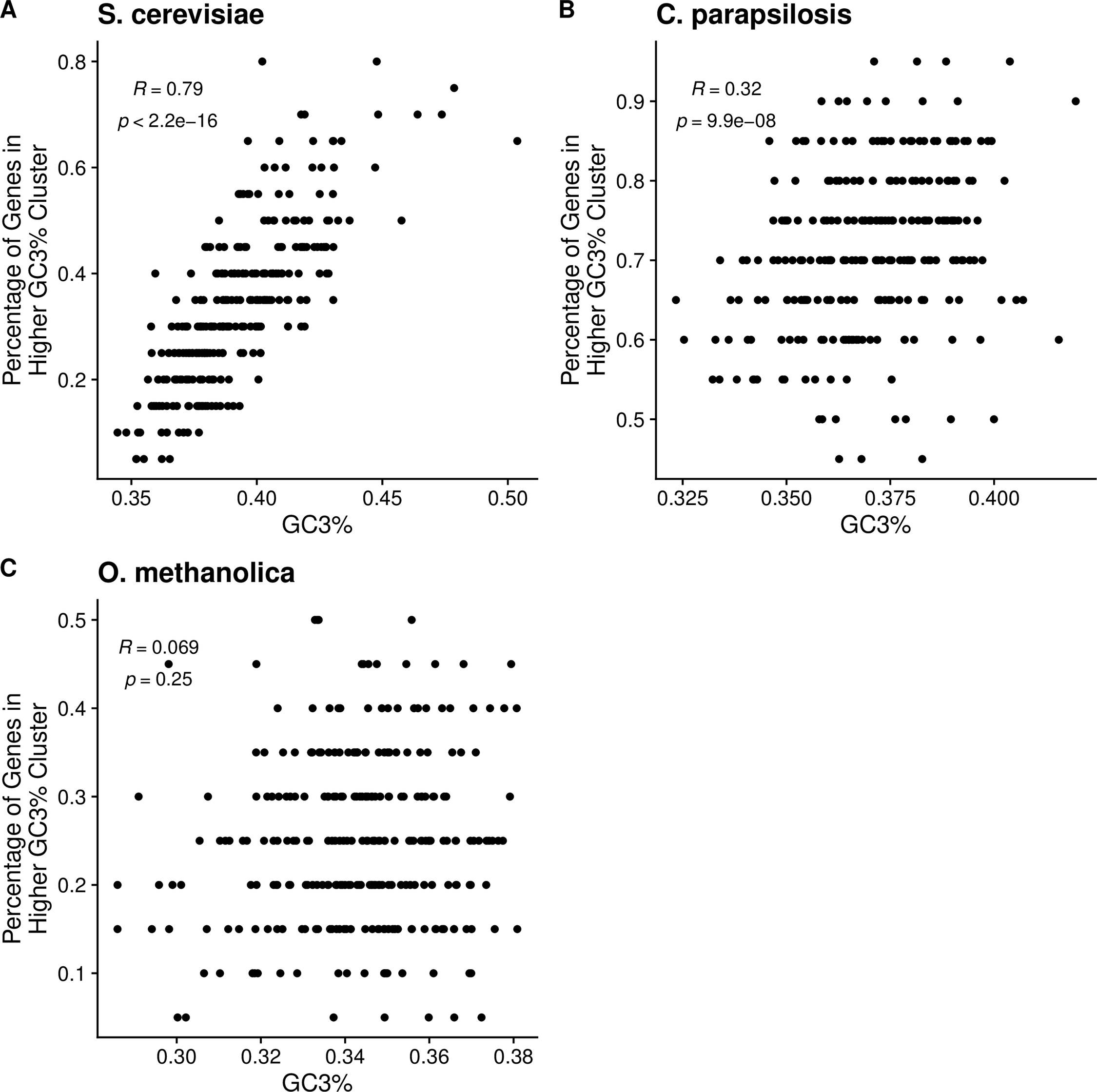

Supplement: S16 Fig — Comparing the average GC3% and percentage of genes assigned to Higher GC3% cluster using 20-gene non-overlapping windows across all chromosomes for a species. (A) S. cerevisiae. (B) C. parapsilosis. (C) O. methanolica. (TIF) [file pgen.1010256.s016.tif]

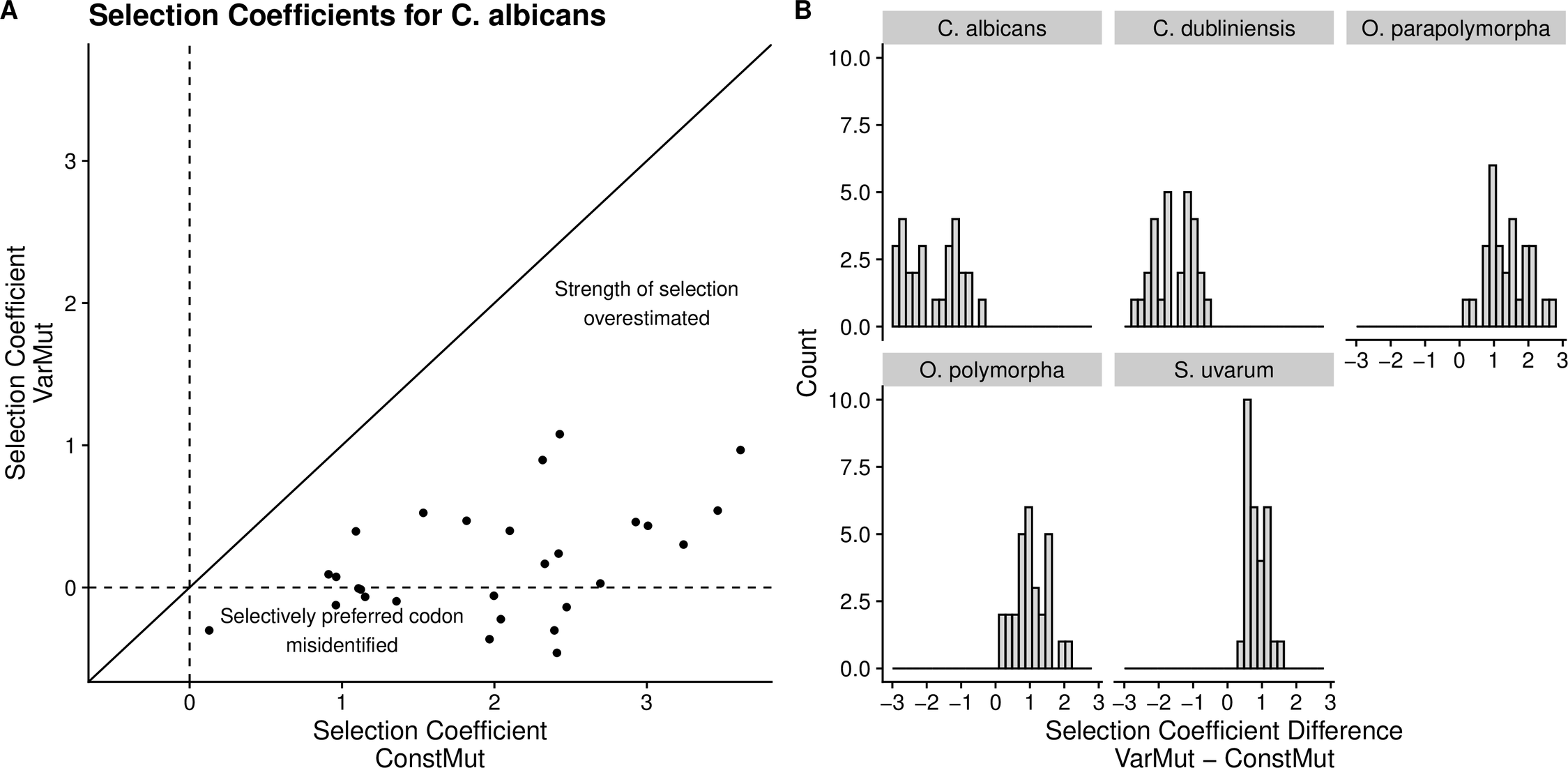

Supplement: S17 Fig — Selection coefficients are modified such that they represent selection of GC-ending codons relative to AT-ending codons, i.e. NNG relative to NNA or NNC relative to NNT. (A) Scatter plot showing the effect of intragenomic mutation bias on misidentifying or overestimating the strength of selection on GC-ending codons relative to AT-ending codons in C. albicans. (B) Distribution of log fold changes of selection coefficients between VarMut and ConstMut models. (TIF) [file pgen.1010256.s017.tif]

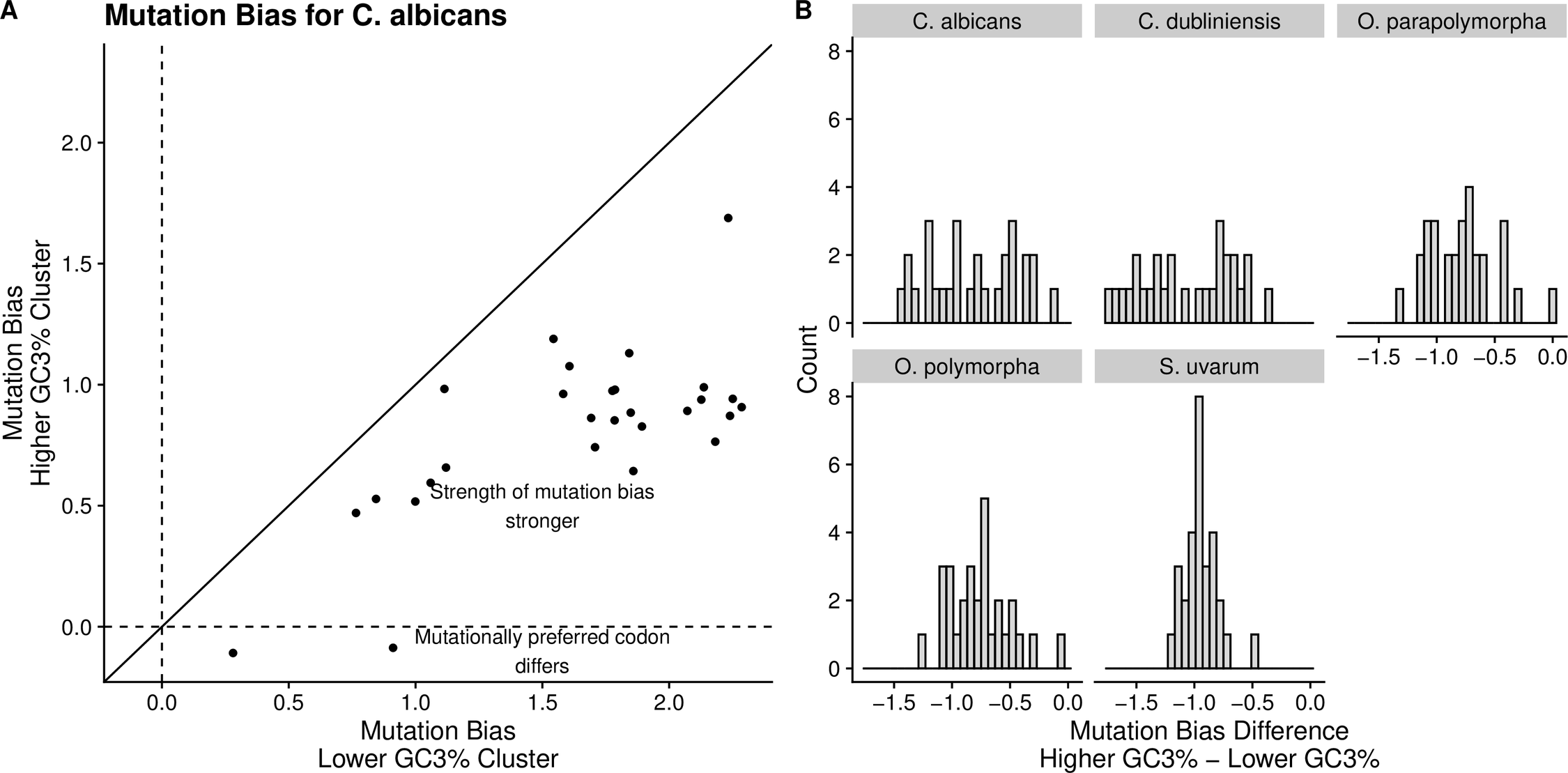

Supplement: S18 Fig — Mutation biases are modified such that they represent mutation bias of GC-ending codons relative to AT-ending codons, i.e. NNG relative to NNA or NNC relative to NNT. (A) Scatter plot showing the difference in mutation bias between the two clusters used in the VarMut model for C. albicans. (B) Distribution of log fold changes of mutation bias between clusters used in VarMut Model. (TIF) [file pgen.1010256.s018.tif]

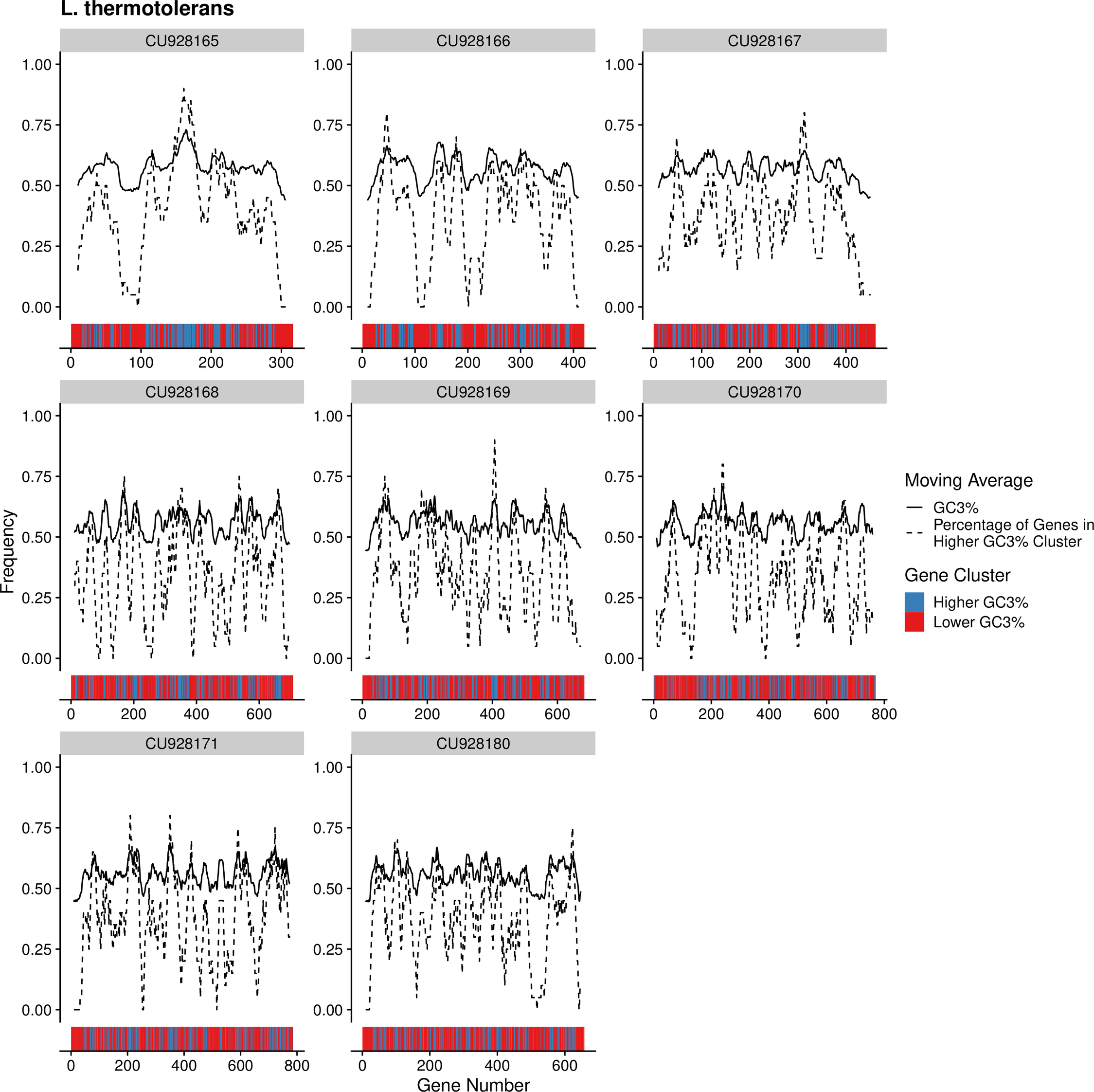

Supplement: S19 Fig — Per-gene GC3% content across all L. thermotolerans chromosomes quantified as a moving average using a 20 gene sliding window (solid line). For each 20 gene window, the percentage of genes assigned to the Higher GC3% regime is also shown (dashed line). Color bars indicate the mutation regimes for Higher and Lower GC3% (blue and right, respectively). (TIF) [file pgen.1010256.s019.tif]

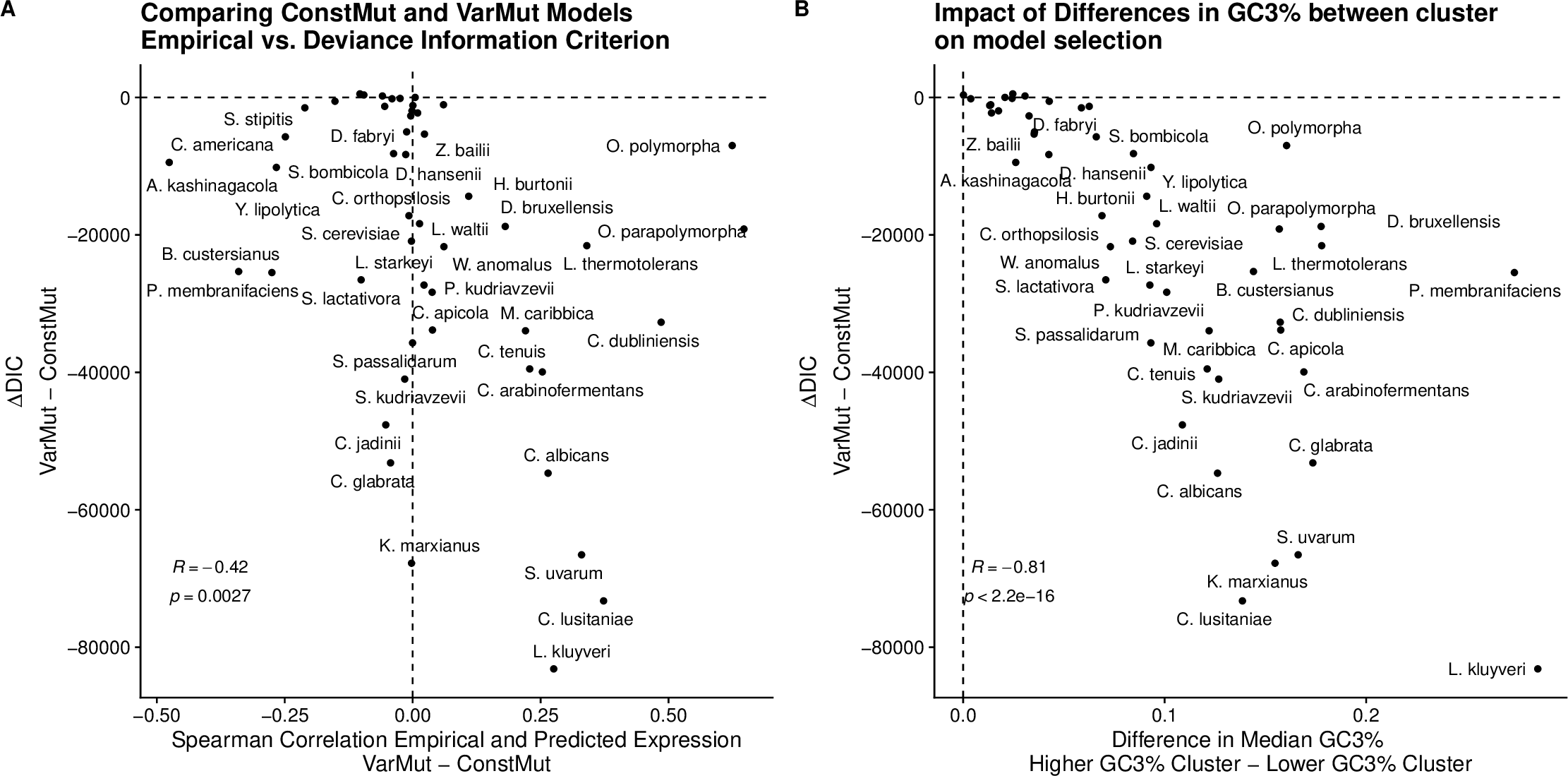

Supplement: S20 Fig — Comparison of ΔDIC to (A) differences in prediction of gene expression data between the VarMut and ConstMut models and (B) the differences of the median GC3% values of the Higher GC3% and Lower GC3% clusters. (TIF) [file pgen.1010256.s020.tif]
