# Supplementary material for: Intragenomic variation in non-adaptive nucleotide biases causes underestimation of selection on synonymous codon usage
Source: PLoS Genet. 2022 Jun 17;18(6):e1010256. doi: 10.1371/journal.pgen.1010256 (PMC9246145; doi:10.1371/journal.pgen.1010256)
Supplement: Attachment — Submitted filename: Cope_and_Shah_Plos_Genetics_Response_to_Reviewers.docx [file pgen.1010256.s022.docx]

**Reviewer #1**: This manuscript analyzes codon usage bias (CUB) in 49 species of yeast, with a focus on the influence of genome-heterogeneous substitution processes. It is argued that a failure to model this heterogenity can lead to misleading inferences regarding the strength of CUB.

I found some of the analyses and results interesting - particularly, the contrasted pattern between closely related species shown in figure 1. I have a number of remarks regarding the writing and the overall organization of the manuscript. Importantly, I feel like the main message of the manuscript is not particularly unexpected. Model misspecification is well identified as an important statistical problem in evolutionary genomics in general, heavily discussed in the molecular phylogenetic and population genomic literature. That this problem can also apply to model-based CUB analysis, as demonstrated here, is not surprising. The manuscript uses a specific method, ROC-SEMPER, which although impressively sophisticated, has not been applied to many data sets so far (28 cites since 2015). It is unclear whether the detected biases would also affect, and how much, other approaches to the analysis of CUB. Finally, the reasons for the discrepant patterns among closely-related species are only superficially considered in the manuscript, while I see this as one of the most intriguing aspects of the study. Below I'm providing a couple of more detailed comments, which might help, I hope, improve the manuscript.

We thank the reviewer for their detailed and thoughtful review of our work. We completely agree with the reviewer that model misspecification is a recurrent problem in many aspects of phylogenetic and population genetics studies. In that sense, what we’ve uncovered here is not terribly surprising. What was surprising, at least to us, was the degree of variation in model fits between closely related species. Moreover, we were able to decouple and identify the source of this variation to be coming from non-adaptive processes. Following reviewer’s suggestion, we have added additional analysis of codon usage patterns using heuristic measures such as Codon Adaptation Index (CAI calculated via CodonW) to show that this problem can impact other metrics as well (Supplementary Figure 2). We discuss these details in depth in relevant comments below.

- Manuscript organization 1

49 yeast genomes are analysed independently using the ROC SEMPER method. The main analysis only considers 8 of these, out of which 5 are problematic. Then, results from the whole 49 are shown, and we get to know that the remaining 41 are not strongly, or at all, affected by the detected bias. I find this way of presenting the data somewhat misleading in leaving the impression, throughout the reading, that the bias affects a large proportion of the analysed species, whereas in reality this proportion is 10%. I see no good justification for such a progressive introduction of the results.

We debated the merits of this alternate organization of the manuscript during its early drafts. We concluded that starting the manuscript with overall analyses of 49 fungal genomes might give the readers a misleading view of the focus of the manuscript. It might suggest that we were going to study how selection on codon usage and gene expression patterns evolve across a phylogeny. We are working on a follow-up manuscript where we perform an in-depth analysis on these topics and demonstrate how phylogenetic constraints help explain many of the observed changes. Nonetheless, following the reviewer's suggestion,we have updated the manuscript to first introduce how the ConstMut model generally does a good job across the 49 species before diving into some of the more problematic cases. We include a new supplementary figure in support of these arguments..

- Manuscript organization 2

Some additional writing effort is needed if the Methods section is to be located after the Results and Discussion sections, as it currently is. A brief presentation of the data and an explanation of the methods should be inserted at the begining of the Results section, which currently is uneasy to follow - the reader needs to go back and forth between Results and Methods. Generally, I think the ROC SEMPER method and underlying model parameters would deserve to be inroduced in more detail.

We agree and have added brief descriptions of the model and approach at the beginning of the specific Results section. We have also significantly expanded our description of ROC-SEMPPR in the Methods sections. We hope this improves the readability of the manuscript.

- Analysis

An unsupervised classificaton method is used to define two groups of genes per species. Is there any particular reason why two groups were chosen, not 3 or more? Is there a way to assess the significance of the partitioning?

The reviewer raises an excellent question about the ideal number of partitions. In the initial stages of the project we did use model selection based approaches to identify the right number of partitions for the data. We now include some of these analyses in the supplement. Briefly, for the five species considered in our analysis, we highlight the effects of using more than 2 clusters. As can be seen in Supplementary Figure 7, 3 clusters usually results in poor(er) ability to predict gene expression from codon usage patterns than using 2 clusters in most cases. This is perhaps not surprising in hindsight because with a higher number of clusters, we run the risk of over-partitioning genes such that it is likely that most or all highly expressed genes might get clustered into a single group. As a result, our ability to separate out the effects of non-adaptive and adaptive forces on codon usage patterns using ROC-SEMPPR diminishes significantly.

The two groups are called low-GC and high-GC, and interpreted throughout as reflecting an inherent within-genome variation in GC-content; so why not simply partition the data based on gene GC3?

We were concerned that *a priori* partitioning genes based on GC3 content alone would lead to partitioning of genes based on their expression levels as GC3 frequency typically tracks expression data. Therefore, we decided to use an unbiased clustering approach using CLARA. The unsupervised learning approach allows the data to “speak for itself” while minimizing *a priori* assumptions. The fact that GC3 content was one of the major determinants (and by no means the only factor) of the two clusters was quite a surprise.

- Interpretation 1

The fact that genes of similar GC-content tend to be clustered is interpreted as supporting the hypothesis that GC-biased gene conversion (BGC) explains the among-genes heterogeneity in GC-content. I am not sure I'm following the logic here. BGC is a recombination-associated mechanism, so I would expect BGC to result in such a clustering if recombination was genome-heterogeneous, and correlated to gene GC-content. Do we have data on the recombination map of some of these species? Or reasons to believe that clusters of GC-rich genes fall in high-recombining regions? Also at odds with this explanation is the fact that BGC is a well documented process in S. cerevisiae (eg Mancera et al 2008 Nature; Harrison & Charlesworth 2011 MBE, Lesecque et al 2013 MBE), but S. cerevisiae is identified as a non-problematic, GC-homogeneous species in this study.

Correlations between GC content and recombination rates have been described previously, such as in the supplemental material in Harrison & Charlesworth MBE 2011. We did observe that recombination hotspots identified in S. cerevisiae were biased towards the coding sequences assigned to the Higher GC3% cluster, but we decided not to include this analysis for two reasons. 1) We have updated the manuscript to take a neutral stance on the potential causes for the observed variation. 2) The recombination hotspots only include a small percentage of coding sequences, so this is still weak evidence, in our opinion.

We note that Harrison and Charlesworth also found that gBGC has a relatively weak impact on codon usage relative to selection in the sensu stricto yeasts, but was strongest in S. uvarum (or S. bayanus). As noted in the Discussion, this seems consistent with our results. However, recent work by Liu et al. Nat. Eco. Evo. 2017 challenges the idea that gene conversion is biased in S. cerevisiae and notes that the apparent relationship between GC content and recombination rates could be due to recombination occurring more frequently in high GC regions (see also Kiktev et al. PNAS 2018).

- Interpretation 2

In one previously-studied species, Lachancea kluyveri, lateral gene transfer (introgression) was identified as the cause of GC-content heterogeneity. The text says: "For example, both L. kluyveri and L. thermotolerans show a substantial improvement in the predictions of gene expression with the VarMut model; however, only the former contains the introgressed region on one of its chromosomes [48]." This is not a correct statement since L. thermotolerans is not analysed in ref 48. Couldn't GC-content heterogeneity be explained by introgression (of smaller pieces) in this and other species as well? Given the phylogenetic proximity of these species to species with a ~homogeneous genome, this would appear as a plausible explnation to me; I do not see why it is disregarded.

We apologize for the lack of clarity in this section. When talking about *L. kluyveri and L. thermotolerans*, we were specifically drawing attention to the large introgression that resulted in the high GC content on the left arm of a single chromosome in *L. kluyveri* . We do not mean to imply that the results in *L. thermotolerans* could not be due to various smaller introgressions. We have edited the text to make this clear. We have also added the references within Landerer et al. BMC Evo. Bio. 2020 to cite the studies that directly studied the Lachancea genus.

- Interpretation 3

The BGC hypothesis is favoured, and sometimes connected to the (in)ability of various species/clades to engage in sexual reproduction, but I must say I found the whole rationale confusing. A better job could be done of informing the reader of what is known and unknown regarding sex in these 49 species, and whether this relates to the observed patterns.

We completely agree with both reviewers’ concerns about linking the observed patterns of clustering of genes to BGC hypothesis. We believe that while BGC might play some role in driving these patterns, given the variation in the degree of sexual reproduction across species and the lack of controlled data to quantify these measurements, it is important to provide a more nuanced argument and present alternate explanations. We have revised the text to address these issues..

- Wording 1

The word "isochores" is used in the introduction to describe the existence of GC-content heterogeneity in some vertebrate and yeast species. I would suggest refraining from using this term as it has a long history of adding confusion to the literature - e.g. see https://en.wikipedia.org/wiki/Isochore_(genetics).

We thank the reviewer for bringing this to our attention. Following their recommendation, we have now dropped the word “isochores” from the manuscript.

- Wording 2

GC-biased gene conversion (BGC) is identified throughout as a mutation bias. This is not correct. BGC is a segregation bias which tends to favour the increase in frequency and eventual fixation of GC alleles in high-recombining regions of the genome. From a population genetic viewpoint, BGC acts similarly to selection, not mutation, although no fitness effect is involved (eg see work by Duret). I understand that the effect of BGC is likely captured by the mutation bias parameters of the ROC SEMPER method, but this is no good reason to call BGC a mutation bias - it is not.

We completely agree with the reviewer and have updated the manuscript to say “nonadaptive nucleotide bias” in place of mutation bias, where appropriate. We also clarify why the effects of gBGC are expected to be absorbed into the mutation bias parameter.

**Reviewer #2**: The manuscript by Cope and Shah performs a series of analyses of codon usage bias in genomes of budding yeast species to show that variation in mutational biases within each genome can lead to underestimation of selection on synonymous codon usage. The manuscript is generally clearly written and to the point and fits well with the scope of PLoS Genetics. The results of the study are somewhat intuitive and the work significantly contributes to our understanding of codon usage bias and of mutational bias in eukaryotic genomes.

We thank the reviewer for their favorable view of our work and its suitability for PLOS Genetics.

I have one major concern. The authors test two different models, one that assumes constant mutation bias across the genome (ConstMut) and one that assumes variable mutation bias across the genome (VarMut). For 5/8 species tested, the VarMut model gives a better fit between empirical and predicted expression estimates, so the authors conclude that it is better than the ConstMut model. However, the VarMut model has more parameters so how do the authors exclude the possibility that this is due to overfitting? My sense is that there is need to formally compare the two models (e.g., using Akaike Information Criterion or some other formal method).

The reviewer is correct that the VarMut model has a greater number of parameters than the ConsMut model and it is possible that the better fit of the VarMut model could have been due to overparametrization. We chose to compare our predictions of gene expression to empirical RNA-seq data because the models never see the RNA-seq data, so any improvements in prediction would not be due to overfitting. However, following the reviewer’s suggestion, we have now included the results from our comparisons of Deviance Information Criterion (DIC), which is a Bayesian model information criterion. We chose this metric because it is relatively easy to compute from a Markov Chain Monte Carlo simulation. Like AIC, the model with the minimum DIC value is the “better” model.

We find that the difference between DIC (calculated as DIC_VarMut - DIC_ConstMut, 𝛥DIC) was negatively correlated with the difference between models of the Spearman correlations comparing predicted and empirical expression data (calculated as Correlation_VarMut - Correlation_ConstMut, same as in Figure 5). This is consistent with expectations, i.e. a more positive shift in the correlations resulted in more support for the VarMut Model (i.e. a negative 𝛥DIC). Interestingly, many species with very small shifts in the correlations (either positive or negative) clearly favored the VarMut. This could be due to DIC often favoring overfit models (the penalty for overparametrization is not strong) or due to other limitations of our approach, which we explain in the Discussion section.

Minor comments:

- A table that reports the accession numbers of all gene expression datasets of the 49 yeast species used in this study should be provided.

We now include a supplementary table (Supplementary Table 1) with the accessions for each RNA-seq measurement used in the manuscript.

- I spotted quite a few typos (e.g., line 54: "translational", line 118: "suggest", meaning of line 185 not clear, etc.), so some careful checking will be necessary.

We have now fixed these typos and proofread the manuscript to remove any other typos.

**Reviewer #3**: In the manuscript entitled « Intragenomic variation in mutation biases causes underestimation of selection on synonymous codon usage » Alexander L Cope and Premal Shah demonstrate that biases towards GC can be misinterpreted as positive selection on codon usage in yeasts.

I found the manuscript interesting. My main criticism concerns the writing of the material and methods that did not help me to understand what has been done.

We thank the reviewer for their detailed and thorough comments. We have significantly expanded the Methods section of the manuscript to now include details about the underlying ROC-SEMPPR model as well. We have also included additional details about the methods before each of the results section to improve the readability of the manuscript.

MATERIAL AND METHODS

- How many genes do you have per species ?

The number of genes vary widely across the species considered here. There are between 4,200 to 8,700 protein-coding sequences.

- please describe the unsupervised learning method from [52] in one or two extra sentences.

We have further described the correspondence analysis and CLARA clustering in the Methods section.

- please define RSCU as you use it at some point (supp fig 2) even if you don’t use it for CA.

We have defined RSCU in the Methods section. RSCU stands for Relative Synonymous Codon Usage (Sharp et al. Nuc. Acids. Res. 1986) and is a measure of the frequency with which different synonymous codons are used in a gene/genome relative to the frequency expected under a uniform/unbiased distribution.

- please describe the CLARA algorithm a little bit more in the material and metods (at the moment the description is in the results section)

We have further described the correspondence analysis and CLARA clustering in the Methods section.

- please describe in one or two more sentences what ROC SEMPPR does to facilitate the reading. For instance I don’t get the link between the exclusion of CTG codon (serine) and the weak selection hypothesis even if I get that you exclude it because in some species CTG does not translate to serine. Is it really that ROC SEMPPR only assumes weak selection or is it an assumption of codon usage ?

We have generally tried to expand on the details of ROC-SEMPPR in both the Results and the Method sections. We apologize for the confusion regarding “weak selection” and “weak mutation” aspects of our models. Selection on codon usage is thought to be relatively weak (i.e. $N_es << 1$) with genome-wide codon frequencies at selection-mutation-drift equilibrium (Li Journal of Mol. Evol. 1987, Bulmer Genetics 1991, Shah and Gilchrist PNAS 2011). However, “weak selection” has nothing to do with the exclusion of CTG codon in our analysis.

The population genetics framework used in our model is based on the Sella and Hirsh approximation (Sella and Hirsch PNAS 2005) of the Wright-Fisher process and the model assumes “weak-mutation”. Under the “weak-mutation” regime, the population is assumed to be monomorphic for each allele i.e. each mutation get either fixed in a population or is lost prior to the arrival of the next mutation. A constraint of the weak-mutation model is that you can allow mutations only between one-step mutational neighbors of synonymous codons. As a result, unlike Leucine or Arginine where there exists at least one mutational path connecting all 6 of their respective codons, we already treat the 6 serine codons as two separate amino acids as the groups of 4 and 2 codons are two mutations away from each other. Now, in genomes with recoding of CTG codon to serine, this creates a third group since the CTG codon is at least two mutations away from the other two groups. We have clarified this in the Methods section.

line 349 : you say that you scale the codon usage. Is it GCG/GCA or (GCG -GCA)/GCA ? Please describe precisely tha scaling method.

We apologize for the confusion and we hope our further descriptions of ROC-SEMPPR are informative. The selection coefficients and mutation biases estimated by ROC-SEMPPR are relative to a reference synonymous codon that is arbitrarily chosen to be the alphabetically last synonymous codon for an amino acid. When we say rescale these parameters, we are referring to changing the reference codon. As all parameter estimates for a set of synonymous codons are already relative to the same reference codon, the rescaling is done simply by subtracting the parameter estimates (e.g. GCG - GCA). Note that we have updated the manuscript to say we “modified” the parameter estimates in order to change the reference codons. We also explicitly state that this is accomplished via subtraction of the parameter estimates.

-please mention somewhere what are the 49 species.

We have added a supplementary table (Supplementary Table 1) listing all the 49 species considered here. The names are also present in Fig. 5.

RESULTS

-line 101 : it seems that S. cerevisiae is the outlier species but there is also an other species at 0.42 that could be an outlier. What is the statistical test that lead to the outlier/not-outlier classification ?

To clarify, each genera (*Saccharomyces, Candida, and Ogataea*) represented in Figure 1 has one species that we think the ConstMut model is reasonably capturing selection on codon usage, as evidenced by a moderately strong relationship between predicted and empirical expression. The expected result for other species should look similar to those of *S. cerevisiae, C. parapsilosis, or O. methanolica*, but that’s not what we see for the other species.

- line 116-119 : I don’t see the link between the supp fig 3 and the text : are they the same codons accross species ?

We apologize for the confusion. This figure (now Supplementary Figure 4) compares the RSCU values for all 61 sense codons calculated from high expression genes for each species within the same genus.

- line 125 : epigenetics could also lead to differential possibility of mutations

This sentence has been deleted from the manuscript.

-line 140: super interesting !! great to read!

We appreciate this comment from the reviewer.

- line 149-151:you directly link expression prediction and selection. Please rephrase to explicit the step taken here.

We have tried to make this sentence clearer.

-figure 2: what does each point represent?

Each point represents a different gene, comparing its empirical mRNA abundance (from RNA-seq) and its predicted gene expression from ROC-SEMPPR. We have tried to clarify this in the figure captions.

- line 163 : which figure ?

We apologize for the confusion. This sentence is just a general description of what we did. The relevant figures are referenced in the next paragraph (Figure 3 and Supplementary Figures 8 – 15).

- Figure 3 : I don’t know the proportion of genome that is outside genes. If sufficient, it would be interesting to compare GC3 content to GC content outside genes as gBGC should affect similarly GC3 (under weak selection) and GC outside genes.

We have updated the manuscript to take a neutral tone towards the potential biological causes of the observed variation. Even so, we’re not sure this would support gBGC over some of the other hypotheses discussed in the manuscript, such as the variable concentration of G and C nucleotides during S phase.

- Figure 5A : how can you be less good with more parameters (you can not improve the predictions but how can you decrease the correlation ? I think I miss what is a correlation shift. Again the material and methods must be expanded.

We apologize for the confusion. The correlations in question are the correlations between empirical gene expression (i.e. from RNA-seq data) and predicted gene expression (i.e. from ROC-SEMPPR). The correlation shift represents the difference in this correlation between the ConstMut and VarMut models. We were also surprised to initially see a negative shift, but we realized that because codon usage also changes with gene expression, clustering could actually be partitioning genes into groups of high and low expression. This appears to be the case for the VarMut fits for C. parapsilosis and O. methanolica (Fig. 2C): for both species, one cluster contains all of the high expression genes. As ROC-SEMPPR estimates selection and mutation bias parameters by accounting for the natural variation in gene expression, partitioning genes based on gene expression could actually weaken signals of selection and mutation bias, leading to overall worse predictions of gene expression. We note that our model fits with ROC-SEMPPR never see the empirical gene expression.

CONCLUSION/DISCUSSION

- Please explicit in further details what it implies with efficiency of gBGC or other mechansisms such as mutational biases and the fistinction between VarMut and ConstMut models per species. Is there gBGC in S.cerevisiae and in the 7 other species when they Var and Const agree/disagree etc.

It is hard to say given our current knowledge of these processes in many of these species. To the best of our knowledge, S. cerevisiae is the only yeast under consideration for which gBGC has been directly observed, and even that has recently been brought into question by Liu et al. Nat. Eco. Evo. 2017. We do remark that the apparent discrepancy between S. cerevisiae and S. uvarum are consistent with the findings of Harrison and Charlesworth Mol. Biol. Evol. 2011, in which they found gBGC to be more efficient in the latter species. We have revised the manuscript to take a more neutral stance on the potential causes of the observed patterns.

GENERAL COMMENTS

- I am always confused when authors say that gBGC is a mutation bias. gBGC does not create new mutations but is rather a fixation/substitution bias. When there is a GC in front of a AT, gBGC occurs and tends to favor the spread of the GC polymorphisms up to fixation. To that respect I would rephrase the entire manuscript (it does not affect the global conclusion that gBGC affect the efficacy of positive selection identification).

We have updated the manuscript to refer to variation in non-adaptive nucleotide biases, as opposed to mutation biases. We note in the Results section that the effects of gBGC are expected to be absorbed into the mutation bias parameters because it is not expected to covary with gene expression.

- I am missing some inter species comparisons. There must be genes highly express in all the species (housekeeping genes) → do they have the same codon usage/ constraints in GC content ? I ask this question because you mention several times that close species have different GC-constraints, I wonder whether it concerns the entire genomes or solely the species-specific highly express genes. I think this would be an interesting analysis to perform if it does not take too much time.

Even though different species may have different GC constraints, the direction of selection on codon usage (i.e. which codons are favored) is usually determined by the tRNA pool. We have an upcoming manuscript that focuses more in-depth on the evolution of codon usage across species, which includes these and other budding yeast species. We note that Supplementary Figure 4 compares the RSCU values of codons estimated from empirically-determined highly expressed genes across closely-related species. As can be seen in these figures, these RSCU values are moderately to strongly correlated, which suggests the direction and nature of selection on codon usage is mostly consistent across these species.

- You barely mention recombination in your paper even if you discuss a lot gBGC. If it is not too difficult, it would be super interesting and would help validating your work to compare in S.cerevisiae the localisation of the high-GC3 clusters to its recombination map (from Mancera for instance but there are more recent work probably). You could check whether high-GC3 clusters are in high recombining region and thus, under strong gBGC.

We have updated the manuscript to take a more neutral tone towards the possible mechanisms causing the variable non-adaptive mutation biases. We do not believe we have evidence to make statements about the specific causes of the intragenomic variation in non-adaptive nucleotide biases. We did find that the recombination hotspots identified for S. cerevisiae in Mancera et al. were heavily biased towards the High GC3% cluster, but this was also a very small proportion of genes. We decided not to include this analysis because we are trying to take a neutral position on the biological causes and the relatively small proportion of genes that fall into the recombination hotspots is still fairly weak evidence, in our opinion.

- I would emphesize more the unsupervised clustering approach, I think this is great and a real plus-value of your work.

We would like to thank the reviewer for this comment. We have provided more details about the algorithm and how it works.

I did not check the english spelling as it is not my maternal langage.

References

1) Bulmer M. The selection-mutation-drift theory of synonymous codon usage. Genetics.570

1991;129.

2) Harrison RJ, Charlesworth B. Biased Gene Conversion Affects Patterns of Codon Usage and

Amino Acid Usage in the Saccharomyces sensu stricto Group of Yeasts. Molecular Biology

and Evolution. 2011;28:117–129. doi:10.1093/MOLBEV/MSQ191

3) Landerer C, O’Meara BC, Zaretzki R, Gilchrist MA. Unlocking a signal of introgression from

codons in Lachancea kluyveri using a mutation-selection model. BMC Evolutionary Biology.

2020;20:109. doi:10.1186/s12862-020-01649-w.

4) Lesecque Y, Mouchiroud D, Duret L. GC-biased gene conversion in yeast is specifically associated with crossovers: Molecular mechanisms and evolutionary significance. Molecular Biology and Evolution. 2013;30:1409–1419. doi:10.1093/molbev/mst056.
5) Li WH. Models of nearly neutral mutations with particular implications for nonrandom

usage of synonymous codons. Journal of Molecular Evolution 1987 24:4. 1987;24:337–345.

doi:10.1007/BF02134132

6) Liu H, Huang J, Sun X, Li J, Hu Y, Yu L, et al. Tetrad analysis in plants and fungi finds

large differences in gene conversion rates but no GC bias. Nature Ecology and Evolution.

2017;2:164–173. doi:10.1038/s41559-017-0372-7

7) Mancera E, Bourgon R, Brozzi A, Huber W, Steinmetz LM. High-resolution mapping of

meiotic crossovers and non-crossovers in yeast. Nature 2008 454:7203. 2008;454:479–485.

doi:10.1038/nature07135.

8) Sella G, Hirsh AE. The application of statistical physics to evolutionary biology. Proceedings

of the National Academy of Sciences of the United States of America. 2005;102:9541–9546.

doi:10.1073/pnas.0501865102

9) Shah P, Gilchrist M. Explaining complex codon usage patterns with selection for translational543

efficiency, mutation bias, and genetic drift. PNAS. 2011;108:10231–10236.

10) Sharp PM, Tuohy TMF, Mosurski KR. Codon usage in yeast: cluster analysis clearly

differentiates highly and lowly expressed genes. Nucleic Acids Research. 1986;14:5143.

doi:10.1093/NAR/14.13.5125
