# Supplementary material for: Intragenomic variation in non-adaptive nucleotide biases causes underestimation of selection on synonymous codon usage
Source: PLoS Genet. 2022 Jun 17;18(6):e1010256. doi: 10.1371/journal.pgen.1010256 (PMC9246145; doi:10.1371/journal.pgen.1010256)
Supplement: Attachment — Submitted filename: Cope_Shah_Response_to_Reviewers_second_round.docx [file pgen.1010256.s023.docx]

We would once again like to thank the editor and the three anonymous reviewers for their helpful feedback that has substantially improved the clarity of the manuscript.

1. On line 284 you say "we expanded our analysis to 49 species". However, you already mentioned the analysis is of 49 species. It would be better to say you returned to analysis of all 49 species.

Thank you for noticing this. We have updated this sentence to say we “return our focus to the 49 species…”. See lines 284-286.

2. It is understandable, especially given the Liu (2017) paper, that you don't attempt to attribute the %GC content variation to a specific evolutionary force, such as gBGC. While the complications regarding this issue were clear in the reviewer response, I think it is important to relay these to the reader in the discussion as this is a natural question for a reader. Currently, you state that gBGC is present in Sc and reference Liu in parenthesis, and later mention that recombination rates are not available for most species. I would encourage you to more directly address this issue by stating (e.g. around line 351) that it would be hard/complicated to attribute %GC3 to specific biological processes, with Liu paper raising questions about the impact of gBGC, the weak relationship between GC3 (Harrison 2011) and recombination, and the absence of recombination rates for most species.

We have updated the suggested paragraph to better reflect our hesitation in attributing our results to gBGC. See lines 352 – 366. We specifically state that Liu (2017) found no evidence of biased gene conversion in S. cerevisiae, noting that the correlation between recombination rate and GC content could be due to recombination occurring more frequently in GC rich regions, as found by Kiktev (2018).
